# Statistical characterization of urban CO_2_ emission signals observed by commercial airliner measurements

**DOI:** 10.1038/s41598-020-64769-9

**Published:** 2020-05-14

**Authors:** Taku Umezawa, Hidekazu Matsueda, Tomohiro Oda, Kaz Higuchi, Yousuke Sawa, Toshinobu Machida, Yosuke Niwa, Shamil Maksyutov

**Affiliations:** 1 National Institute for Environmental Studies, Tsukuba, Japan; 2 Meteorological Research Institute, Tsukuba, Japan; 3 Global Modeling and Assimilation Office, NASA Goddard Space Flight Center, Greenbelt, USA; 4 Goddard Earth Sciences Technology and Research, Universities Space Research Association, Columbia, MD USA; 5 Faculty of Environmental Studies and Graduate Program in Geography, York University, Toronto, Canada; 6 Japan Meteorological Agency, Tokyo, Japan

**Keywords:** Carbon cycle, Atmospheric chemistry, Environmental monitoring

## Abstract

Cities are responsible for the largest anthropogenic CO_2_ emissions and are key to effective emission reduction strategies. Urban CO_2_ emissions estimated from vertical atmospheric measurements can contribute to an independent quantification of the reporting of national emissions and will thus have political implications. We analyzed vertical atmospheric CO_2_ mole fraction data obtained onboard commercial aircraft in proximity to 36 airports worldwide, as part of the Comprehensive Observation Network for Trace gases by Airliners (CONTRAIL) program. At many airports, we observed significant flight-to-flight variations of CO_2_ enhancements downwind of neighboring cities, providing advective fingerprints of city CO_2_ emissions. Observed CO_2_ variability increased with decreasing altitude, the magnitude of which varied from city to city. We found that the magnitude of CO_2_ variability near the ground (~1 km altitude) at an airport was correlated with the intensity of CO_2_ emissions from a nearby city. Our study has demonstrated the usefulness of commercial aircraft data for city-scale anthropogenic CO_2_ emission studies.

## Introduction

Climate change is considered to be one of the consequences of increased emissions of anthropogenic greenhouse gases during the industrial era, and carbon dioxide (CO_2_) is the dominant contributor to the enhanced radiative forcing caused by anthropogenic long-lived greenhouse gases. Atmospheric CO_2_ mole fraction has increased from ~280 ppm (parts per million) in 1750^1^ to > 400 ppm in recent years^2^ due to the rapid growth of human activities and population since the beginning of the industrial era. About half of the anthropogenic CO_2_ emissions related to fossil fuel combustion and human driven land-use change is taken up by the ocean and the terrestrial biosphere^3,4^. In a top-down approach using atmospheric transport models, the global fossil fuel CO_2_ emissions have often been presumed to provide good estimates of the strength of the land and ocean sinks^5^. But recent studies suggest that uncertainties in fossil fuel emission database could lead to significant biases in the optimized estimates of biospheric flux^6,7^. In fact, uncertainties of fossil fuel CO_2_ emission estimates are growing because of increasing contributions from developing countries^3,4,8,9^.

About 70% of the current anthropogenic CO_2_ emissions is considered to come from urban areas that contain over 50% of the world population^10^, and thus accurate quantification of CO_2_ emissions from urban areas is of particular importance. The existing and projected rate of urbanization is different for different parts of the world; many cities in Asia and Africa are expected to continue to see rapid growths in population while cities in developed countries have basically already stabilized^11^. For effective mitigation actions against climate change, various independent approaches must be used to reduce the uncertainties associated with citywide greenhouse gas emissions estimates, and one of those approaches can be provided by atmospheric CO_2_ observations^12^. For this purpose, atmospheric CO_2_ measurements focusing on urban areas have been examined in recent years by means of citywide *in-situ* ground measurement networks^13–16^ or satellite measurements^17,18^. The former methodology provides dense and accurate data and the latter broad spatial coverage, whilst both also have limitations.

Since 2000s, atmospheric observation instruments onboard commercial airliners have successfully acquired extensive number of trace gas (including CO_2_) data^19–21^. The CONTRAIL (Comprehensive Observation Network for TRace gases by AIrLiners) program, an ongoing project that measures atmospheric CO_2_ and other trace gases using aircraft of Japan Airlines^20^, has obtained thousands of vertical profiles of CO_2_ over many airports since 2005^22–25^. Given that major airports are often located reasonably close to large cities and airlines optimize their flight destinations with priority on connecting between large cities, data collected by commercial airliners might provide useful information that can contribute to better assessments of greenhouse gas emissions from cities, complementing ground and satellite measurements. Here we present some statistical results from analysis of urban CO_2_ emission signals contained in vertical profiles of atmospheric CO_2_ data over airports. We present detailed analysis of data obtained at Moscow, Tokyo and 34 other airports, to offer a conceptual framework in which urban emission estimates can be obtained consistently with the statistical properties of the measurements. We show that the magnitude of atmospheric CO_2_ variability increases with intensity of CO_2_ emissions from the neighboring cities, demonstrating the usefulness of commercial aircraft-based measurements for urban emission studies.

## Results

### Moscow

**Figure 1:**
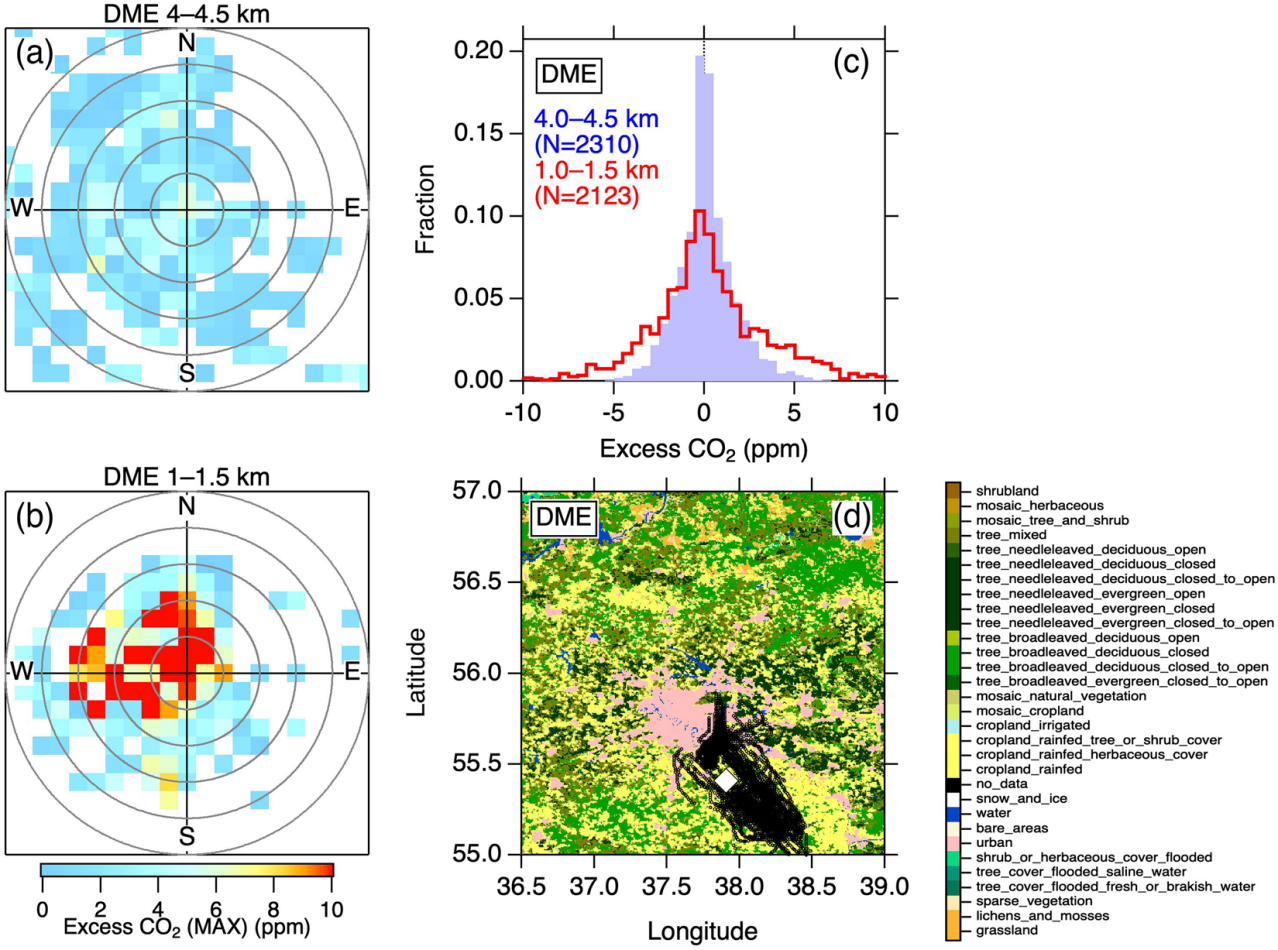
Variability of atmospheric CO_2_ enhancement over Moscow Domodedovo Airport (DME). (**a,b**) Maximum excess CO_2_ values observed in wind direction (angle) and speed (distance from the center) bins at 4.0–4.5 km and 1.0–1.5 km altitude (a.g.l.), respectively. Circles in grey represent every 5 m s^−1^ wind speed. (**c**) Histograms of excess CO_2_ at 4.0–4.5 km (solid purple) and 1.0–1.5 km (solid red line) altitudes. Note that excess CO_2_ = 0 means that the CO_2_ data point lies close to the climatological seasonal variation over the airport, whereas large deviations indicate excursion from the representative seasonal variation. (**d**) The CO_2_ measurement positions at altitudes of <2 km (black circles). The open diamond is the location of DME. The land cover data are from a global land cover map product^36^ and the map was generated by Igor Pro 7 (https://www.wavemetrics.com).

We analyzed appearance of high CO_2_ events in two altitude layers (4.0–4.5 km and 1.0–1.5 km) over Moscow Domodedovo Airport (DME) as a function of wind direction and speed (Fig. 1a,b). These plots indicate wind direction and speed in which high CO_2_ events were observed in the measurement area. At altitudes of 4.0–4.5 km, almost no CO_2_ enhancements in any wind direction and speed are evident (Fig. 1a), indicating that variability of CO_2_ enhancement (defined as excess CO_2_; see Methods) is small in the free troposphere. In contrast, at 1.0–1.5 km, it is seen that high CO_2_ events were associated with winds mainly from the northwest sector (Fig. 1b). At altitudes of < 2 km, our measurement aircraft was primarily in the southeast of the Moscow metropolitan area (pink area in Fig. 1d) in all cases and influenced by CO_2_ emissions from the city when advected from northwest. In addition, the fact that high CO_2_ events appeared with relatively low wind speed (<15 m s^−1^) suggests that a presence of strong emissions close to the airport is likely. The Moscow metropolis covers an area of ~50 km in the northwest-southeast direction, a distance an air parcel with a speed of ~15 m s^−1^ traverses in ~1 hour. Longer residence time of an air mass over the metropolitan area would allow city emissions to be more integrated into the air mass, leading to results consistent with the observations of high excess CO_2_ at low wind speeds. Histograms of excess CO_2_ values at both altitudes (Fig. 1c) shows that the excess CO_2_ at the lower altitude undergoes higher variability (i.e. larger spread of the histogram), and it is likely that the urban plumes contributed to the increased CO_2_ variability with decreasing altitude.

### Tokyo

**Figure 2:**
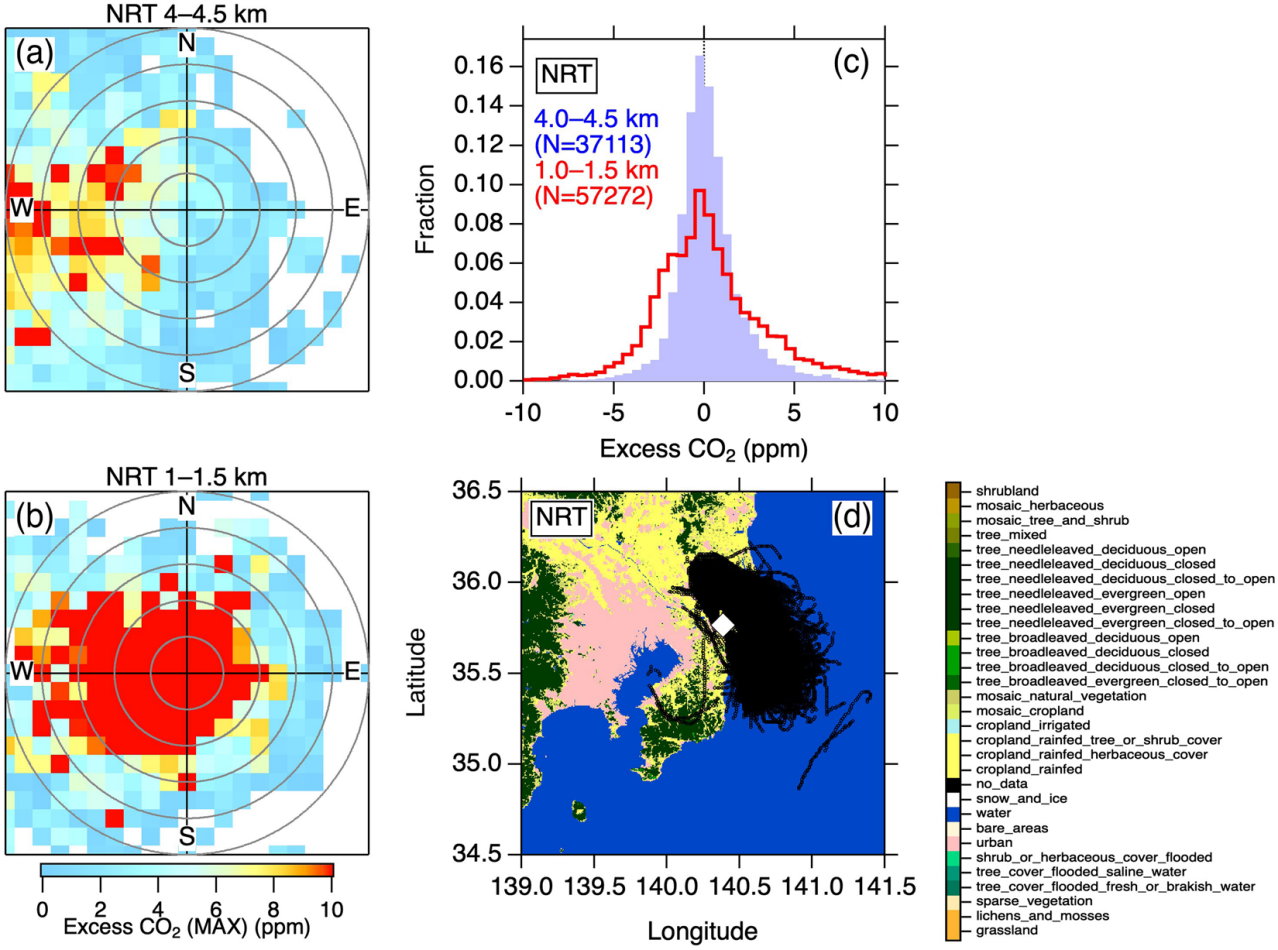
Same as Fig. 1, but for Tokyo Narita Airport (NRT).

Similarly, we analyzed the data over Tokyo Narita Airport (NRT), located ~60 km to the east of the Tokyo agglomeration center (Fig. 2). The flight tracks at <2 km altitudes over NRT are roughly located on the eastern edge of the metropolitan area (Fig. 2d). We found that high CO_2_ air masses were associated primarily with air parcels with relatively weak wind speed from the west of the measurement positions (Fig. 2b). The high excess CO_2_ values (>10 ppm) were associated with <20 m s^−1^ westerly and <10 m s^−1^ easterly. The Greater Tokyo Area is ~100 km wide, the distance a westerly air mass at <20 m s^−1^ would pass over in > ~1.5 hour. Along with the DME analysis, this result suggests that an adequate residence time of >1 hour over the metropolitan area provides measurable accumulation of CO_2_ up to ~1 km altitude in the overlying air mass. At the higher altitudes (Fig. 2a), transport of high CO_2_ was observed in the westerly at relatively high wind speed (>10 m s^−1^). It is again seen that the variability of excess CO_2_ is larger in the lower layer (Fig. 2c).

### Cities worldwide

We applied the above analysis to all of the 36 airports worldwide. At ~1 km altitude over many airports, we identified characteristic wind direction sectors where high CO_2_ events were observed, and in each case the wind direction corresponded well to the geographical location of the urban agglomeration of the nearby large city (Supplementary Fig. S3–S36). High CO_2_ enhancements were usually observed at relatively low wind speeds (roughly < 15 m s^−1^). Vertical distributions of excess CO_2_ indicate that, in most cases, the ~4 km altitude is above the boundary layer, and thus the associated CO_2_ histograms reflect the free tropospheric conditions. In contrast, the histograms in the 1.0–1.5 km layer show considerable broadening at many airports, indicating the presence of nearby surface fluxes that cause fluctuations in CO_2_ in the overlying atmosphere. Therefore, at most airports, the lowermost altitude layers of the CONTRAIL measurements are under substantial influence of CO_2_ emissions from the nearby urban area.

The above results point to the possibility of a relationship between the magnitudes of atmospheric CO_2_ variability and of associated urban CO_2_ emission. We used standard deviation (SD) of excess CO_2_ as an indicator of the magnitude of the short-term CO_2_ variability observed at all airports (Fig. S2). We observed SD exceeding 5 ppm at heights 1 km and lower over several airports, but at every airport, it decreases to <2 ppm at higher altitudes, i.e. the free troposphere (Fig. S37). Although the SD varies seasonally at heights less than or around 1.0 km over some airports (e.g. NRT), the seasonality in the SD is not common over most airports. For the data analysis below, we therefore calculated the annual SD values, regardless of the data gaps at individual airports.

**Figure 3:**
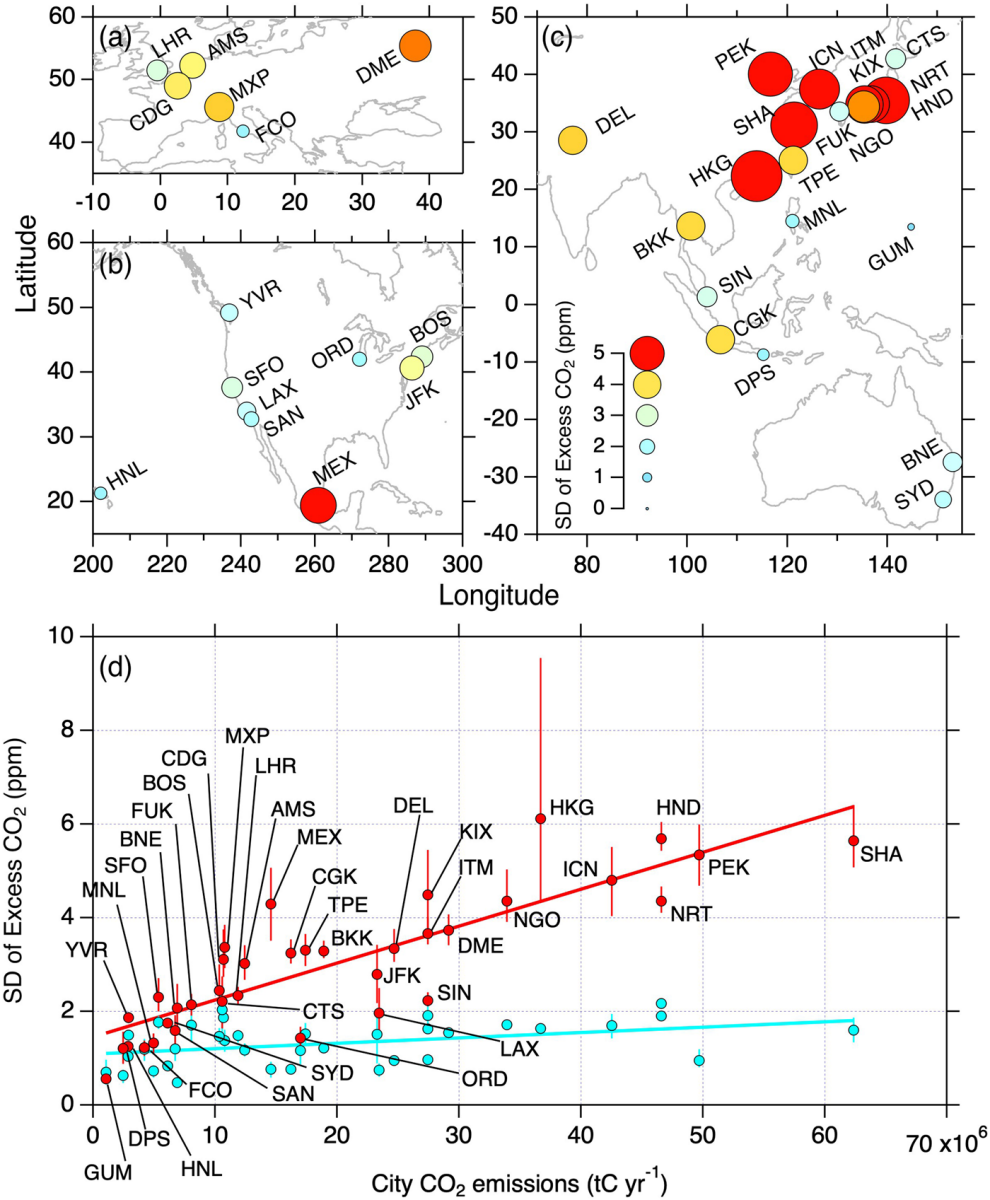
Variability of CO_2_ enhancements over cities worldwide. (**a**–**c**), Maps of the SD values at 1.0–1.5 km altitudes. Size and color of the circles indicate magnitude of the SD (see the legend in panel c). (**d**) Relationship of the SD at 1.0–1.5 km (red) and 4.0–4.5 km (light blue) altitude bins with city CO_2_ emissions based on the ODIAC dataset^26,27^. Solid lines are least square fits to the data at respective altitude bins. Airport codes are indicated for the data from 1.0–1.5 km. The error bars represent range of the SD calculated by a Bootstrap method (*N* = 1000).

We found that CO_2_ variability at 1.0–1.5 km altitudes is largest over some Asian cities, and moderately large over some cities in Europe and North America (Fig. 3a–c). Large CO_2_ variability is associated with urban areas with largest populations in the world; for instance, Tokyo (corresponding airports are NRT and HND) is ranked as the world largest urban agglomeration by population in 2010, and Delhi (DEL), Mexico City (MEX), Shanghai (SHA), and Osaka (ITM and KIX), over which we found the large SD values, are all listed in the top 10 population cities^11^. The most airports with small SD and vertical gradient are located in coastal areas, and the associated cities are relatively small in CO_2_ emissions (Table S1).

We plotted the airport SD as a function of CO_2_ emission from the nearby associated city for 2010 (Fig. 3d). Here we aggregated CO_2_ emissions within 50 km to the east, west, north and south from a representative city center such as city hall, main station and local government building based on the ODIAC (Open-source Data Inventory for Anthropogenic Carbon dioxide, version ODIAC2015) dataset^26,27^. It is seen that, in the free troposphere (4.0–4.5 km), the SD is small (<2 ppm) over all the airports, irrespective of the intensity of CO_2_ emission from a nearby city (*R*^2^ = 0.16); in contrast, at the low altitude (1.0–1.5 km), the SD has a significant correlation with city CO_2_ emission (*R*^2^ = 0.73), suggesting considerable influence of the urban CO_2_ emissions on our measurements. A significant correlation (*R*^2^ = 0.68) is likewise observed for the data below 1 km, but with a larger scatter and a less compact correlation plausibly due to higher variability and less number of data. It is notable that there are some exceptional cities whose variabilities show excursions from the general trend; the SD values over Singapore (SIN), Los Angeles (LAX), and Chicago (ORD) are as small as those in the free troposphere; those over Hong Kong (HKG), Osaka (KIX), and Mexico City (MEX) are disproportionally high with large uncertainty. Here we used the ODIAC dataset, as it provides up-to-date gridded estimates of anthropogenic CO_2_ emissions. It is noted that the use of different anthropogenic CO_2_ emission datasets would not significantly affect the present result, given the fact that the existing CO_2_ emission datasets (including ODIAC), with their major differences originating in the downscaling of national totals, correlate well with each other at the spatial scale of the current study (100 km × 100 km) or even smaller^28^.

Despite various factors that could contribute to the CO_2_ variability over airports, our results strongly suggest that the intensity of the nearby urban CO_2_ emissions is the primary component of the magnitude of the observed CO_2_ variability (Fig. 3d). Our analyses of the wind pattern (Figs. 1, 2 and Supplementary Figs. S3–S36) indicate that the CO_2_ variability at ~1 km altitude is, to a large degree, driven by the advection of high-CO_2_ air masses influenced substantially by CO_2_ emissions from the nearby city. It is likely that larger CO_2_ emissions enhance contrast of atmospheric CO_2_ mole fraction between their downwind and other locations, which makes fluctuations observed during multiple flights larger. The observed correlation may suggest that our lowest measurable altitude (1.0–1.5 km) is plausibly the height region where city-scale (~tens of km) emission imprint is distinct, and in which smaller-scale heterogeneity due to spatial pattern of emissions and local meteorology is relatively smoothed out. Previously, ground measurements in Paris showed that more urbanized part of the city showed larger CO_2_ variations due to spatiotemporal variations of nearby anthropogenic emissions^15^. Moreover, the present result is consistent with a previous modeling study^23^, which showed, by analyzing the CONTRAIL CO_2_ vertical profiles over NRT, that the magnitude of the atmospheric CO_2_ variability in the boundary layer is highly sensitive to the magnitude of anthropogenic local fluxes.

## Discussion

**Figure 4:**
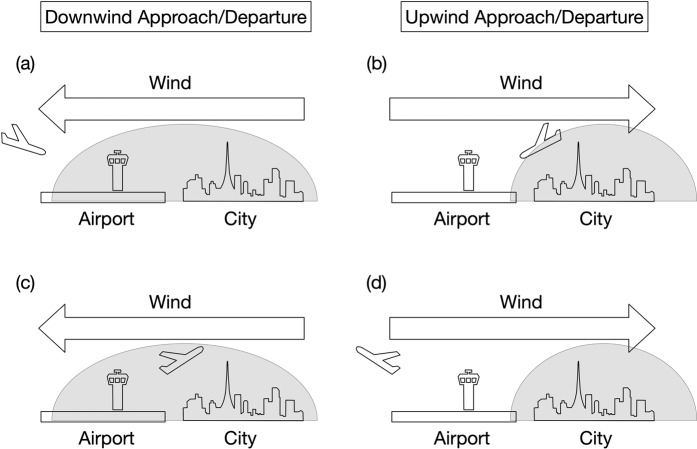
Schematic sketches of aircraft approach/departure under different wind directions. Degree of detection of urban CO_2_ emissions depends on various factors such as location of the airport with respect to the city, wind direction and speed, and development of urban CO_2_ dome/plume (gray shade) around the city.

If we assume constant height of the convective boundary layer, one would expect a linear relationship between the surface CO_2_ emission and the enhancement of the CO_2_ mole fraction in the downwind boundary layer. In reality however, such emission-mole fraction relationship is complicated by the boundary-layer dynamics which undergoes significant daily and seasonal variations, causing fluctuations in the emission-driven CO_2_ accumulation in the boundary layer. Our vertical CO_2_ measurements were made under such varying meteorological conditions, likely a contributing factor in the variability in the SD values around the red line in Fig. 3d. In general, every airport has at least two opposite approach/departure directions along the runway, depending on the wind direction; flight routes are determined by airspace over the airport as well as by meteorological condition, thereby differ from flight to flight. We note that our observations might have been biased by specific flight routes under prevailing wind direction. The CO_2_ mole fraction would be elevated in downwind of a neighboring city when the aircraft enters “urban CO_2_ dome/plume” (Fig. 4a.c). In contrast, when the aircraft is positioned at upwind of the city, CO_2_ emissions from the city would be hardly captured, except in cases where the aircraft flies down over the city (Fig. 4b,d). The varying spatial extent of the CO_2_ dome/plume is another crucial factor in detecting urban emission signals. It is influenced by wind direction and speed, as well as the development and decay of convective boundary layer at the time of observation^15,24^. In this respect, the local time of the measurements is relevant, as commercial aircraft are scheduled at specific time of a day. Likewise, upwind biospheric CO_2_ fluxes that undergo significant diurnal and short-term (shorter than seasonal) variations could contribute to the observed variability. Relative contributions of these various factors would be dependent on the measurement location, which might contribute to the variability around the linear trend in Fig. 3d. Case studies dedicated to data analysis of these contributing factors are required to identify the nature and extent of influence of the local surface fluxes to the observed variations; our earlier work provides an example for Delhi (DEL), India^24^.

The variability-emission relationship in CO_2_ (Fig. 3d) could not have been found without the commercial airliner in-flight measurements that enable worldwide vertical CO_2_ scanning over large cities. We note that the relationship represents atmospheric CO_2_ variability obtained from the last 10 years of aircraft data plotted against the 2010 CO_2_ emission data. Given the above consideration (Fig. 4), it is reasonable to assume that this relationship would hold for other years of city emission data, and further analyses based on yearly or monthly time scale are required to establish this relationship on a much firmer ground. Currently the vertical profile data over Tokyo (NRT and HND), where the measurement is most frequent, might satisfy such requirement. The value of the results (Fig. 3), as interpreted within our conceptual frame (Fig. 4), becomes more obvious in contributing towards constraining anthropogenic CO_2_ emissions for cities in developing countries where uncertainty of emission inventory is large and atmospheric measurements available for top-down emission estimation are sparse.

We also explored the possibility of a relationship of vertical gradient (i.e., CO_2_ difference between ~1 km and the free troposphere) against city CO_2_ emissions, with a hypothesis that strong ground emissions would elevate the near-surface CO_2_ levels while the free troposphere is generally free from surface influence. In other words, vertical CO_2_ gradient could represent the aforementioned linear relationship with associated surface emissions. We found large vertical gradients at some airports with strong nearby CO_2_ emissions, however, we did not find a significant relationship between the vertical gradient and the city CO_2_ emissions for various cities. The calculated vertical gradient was found to be considerably dependent on measurement time, season and location of individual airports. As discussed above, CO_2_ variability over cities is influenced by various factors and, among them, the vertical extent of the CO_2_ dome/plume is of particular importance. It depends on the height of the convective boundary layer that varies seasonally and diurnally and influences the meteorological condition during vertical flight measurements. Seasonal coverage of the data and flight schedule are thus also relevant. Furthermore, geographical location of an airport relative to the nearby city varies significantly from city to city; thus, spatial representativeness of the observed vertical gradient differs among airports. Accordingly, for a global characterization of urban CO_2_ emissions based on vertical aircraft measurements at world’s major airports, as in this study, the vertical gradient would not provide a consistent relationship with corresponding city CO_2_ emissions due to uneven measurement opportunities at various airports. We however consider that it could be a good indicator of CO_2_ enhancement due to surface emissions for in-depth analyses at individual airports.

In summary, this study has demonstrated that the data taken by commercial airliners in airport proximity contain clear advective fingerprints of urban CO_2_ emissions. Better understanding of transport processes of local surface fluxes to the atmosphere for individual measurement locations is also needed. Although the observed variability of CO_2_ in the lower troposphere can be caused by many physical processes, the present study showed that the magnitude of the CO_2_ variability at an airport reflects the amount of CO_2_ emitted into the atmosphere from the corresponding city. The variability-emission relationship deduced from a relatively large number of vertical profile measurements could be helpful for evaluating surface fluxes and vertical propagation processes in the boundary layer simulated by various local to regional scale transport models. Further studies incorporating commercial aircraft data into urban CO_2_ emission studies will contribute significantly towards better estimates of CO_2_ emissions from major cities worldwide. Although vertical profile simulations by a regional transport model have been previously examined with commercial airliner carbon monoxide data^29^, the high-resolution vertical profile CO_2_ data used in this study should be considered for incorporation in urban-scale inversion studies^30,31^ or to be used in an independent validation of the emission estimations derived from ground-based data.

Although some recent projects have focused on urban CO_2_ monitoring and quantification of CO_2_ fluxes from cities, such as Paris^15^, Los Angeles^14^, Indianapolis^13^ and Toronto^16^, we still have long ways to go, particularly when it comes to addressing key upcoming megacities that include those in developing countries^11,12^. In this respect, the advantage of commercial airliner is that measurements could provide a great opportunity to study many cities that have not been probed by the current ongoing urban monitoring projects and contribute to the reduction in uncertainties in the estimates of urban CO_2_ emissions. The present study with Japan Airlines covers many cities in Asia where CO_2_ measurements are sparse and a new set up of secured long-term ground-site measurements is difficult. Although installation of a greenhouse gas measurement system into a European airline is in progress^21^, further implementation, in particular into airlines of the United States, China and United Kingdom whose commercial aviation operations together accounts for about 40% of the world^32^, will significantly extend global monitoring capability of urban areas. Measurements onboard several airlines at same airport will provide vertical profile data at different local-time schedules, which will be also helpful for better quantification of surface fluxes.

## Methods

### Experimental

The CONTRAIL project deploys Continuous CO_2_ Measuring Equipment (CME) and its in-flight measurements started on 5 November 2005. We refer to our earlier papers for details^20,22,25,33^. Briefly, the CME unit measures CO_2_ mole fractions onboard the aircraft using a non-dispersive infrared gas analyzer (LI-840, LI-COR Biogeosciences). As of October 2019, installation of the CME is certified for eight Boeing 777-200ER and two Boeing 777-300ER aircraft of Japan Airlines (JAL). Once installed, the CME is operated automatically using the aircraft’s flight navigation data from the ARINC 429 data bus until it is unloaded from the aircraft about two months later. The measured sample values are compared with two working standard gases (CO_2_ in air) installed inside the CME that are traceable to the NIES (National Institute for Environmental Studies)-09 CO_2_ scale. The NIES-09 CO_2_ scale defines mole fraction of CO_2_ in dry synthetic air in μmol mol^−1^ (reported in ppm in this paper). The results from the Round Robin intercomparison experiment show that the NIES-09 CO_2_ scale differs from the WMO-CO2-X2007 scale (http://www.esrl. noaa.gov/gmd/ccgg/wmorr/wmorr_results.php) by <0.1 ppm. The CME data are recorded at 10-s intervals during ascent/descent (~100-m intervals in altitude) and at 1-min intervals during cruise (~15-km intervals horizontally) as well as in-flight aircraft position, static air temperature and wind data received from the ARINC 429 system. To avoid heavy pollution around airports, CME is not operated within 2000 ft (609.6 m) of the ground surface (this altitude was set to 1200 ft in early years). The analytical precision of the CME is estimated to be <0.2 ppm.

### Data analysis

Here we examined atmospheric CO_2_ variations due to emissions from cities and local surface fluxes. It was assumed that the observed CO_2_ variation is composed of variations on different timescales e.g. interannual, seasonal and shorter-term variations, where local scale signals of our interest most likely contribute to the last term. It is noted that this term also contains synoptic-scale variations which represent signals of larger scale fluxes in space (i.e. regional). We therefore needed to subtract longer-timescale variations according to our earlier analysis^25^ as follows:

1 $$\Delta C{O}_{2}(lat,\,lon,\,alt,\,t)=C{O}_{2}(lat,\,lon,\,alt,\,t)\mbox{--}Trend\,C{O}_{2}\,at\,MLO\,(t)$$

Here *lat*, *lon*, *alt*, *t* are the latitude, longitude, altitude and time of individual CME data points, respectively, and *Trend CO*_2_ *at MLO* is the long-term trend curve calculated from the flask-based CO_2_ data at Mauna Loa (MLO; 19.54°N, 155.58° W, 3397 m.a.s.l.), Hawaii, obtained from NOAA/ESRL/GMD (National Oceanic and Atmospheric Administration/Earth System Research Laboratory/Global Monitoring Division; available at ftp://aftp.cmdl.noaa.gov/data/) with a digital filtering technique^34^. In general, the long-term CO_2_ trend at MLO is representative of the large-scale clean atmosphere and has been used as a reference site; thus, ΔCO_2_ in Eq. (1) gives an estimate of the climatological seasonal variations^25,35^ (see Supplementary Fig. S1). Next we calculated *excess CO*_2_ as follows:

2 $$excess\,C{O}_{2}(lat,\,lon,\,alt,\,t)=\,\varDelta C{O}_{2}(lat,\,lon,\,alt,\,t)-median\,\varDelta C{O}_{2}(airport,\,alt\mbox{--}bin,\,t\mbox{--}bin)$$

Here *airport* refers to the nearby airport where the vertical profile data are taken, *alt-bin* and *t-bin* are the altitude and time-of-the-year bins to which individual data points belong. Individual data points are grouped into 500-m altitude and 14-day bins (giving a total of 26 time bins per year). Note that altitude in this study refers to level above ground of the nearby airport (above ground level, a.g.l.). By subtracting the seasonally varying median values according to Eq. (2), excess CO_2_ represents detrended and deseasonalized variations and is analyzed below in terms of altitude and geographical locations. We also calculated SD of excess CO_2_ in each time bin and found that its seasonality is not common to the data over most airports (Supplementary Fig. S2). For statistical analysis, we selected 36 airports that contained sufficient number of vertical profiles over the 2005–2016 period; the number of vertical profiles ranged from 50 to >7000 (Supplementary Table S1).

## Supplementary information

Supplementary information.

## Data Availability

The CONTRAIL CME CO_2_ data are available on the Global Environmental Database of the Center for Global Environmental Studies of NIES (https://doi.org/10.17595/20180208.001). The data are also available from the ObsPack data product (http://www.esrl.noaa.gov/gmd/ccgg/obspack/) and the World Data Center for Greenhouse Gases (https://gaw.kishou.go.jp/). The ODIAC emission data product is available from the Global Environmental Database operated by the Center for Global Environmental Research, National Institute for Environmental Studies (http://db.cger.nies.go.jp/dataset/ODIAC/).
