# Supplementary material for: Statistical characterization of urban CO2 emission signals observed by commercial airliner measurements
Source: Sci Rep. 2020 May 14;10:7963. doi: 10.1038/s41598-020-64769-9 (PMC7224273; doi:10.1038/s41598-020-64769-9)
Supplement: Supplementary file 1 — Supplementary information. [file 41598_2020_64769_MOESM1_ESM.pdf]

Supplementary material

Statistical characterization of urban CO_2 emission signals observed by commercial airliner measurements

Taku Umezawa[1,*], Hidekazu Matsueda[2], Tomohiro Oda[3,4], Kaz Higuchi[5], Yousuke Sawa[2,6], Toshinobu Machida[1], Yosuke Niwa[1,2], and Shamil Maksyutov[1]

[1]National Institute for Environmental Studies, Tsukuba, Japan.
[2]Meteorological Research Institute, Tsukuba, Japan.
[3]Global Modeling and Assimilation Office, NASA Goddard Space Flight Center, Greenbelt, USA
[4]Goddard Earth Sciences Technology and Research, Universities Space Research Association, Columbia, MD, USA.
[5]Faculty of Environmental Studies, York University, Toronto, Canada.
[6]Japan Meteorological Agency, Tokyo, Japan

*Corresponding Author (umezawa.taku@nies.go.jp)

Supplementary Table S1. List of the airports of the CONTRAIL CO_2 measurements. The column vertical profile shows number of vertical profiles taken over each airport. Flight time is local time. City name is that representative for each airport. Population estimate of each city for 2010 is taken from United Nations [2015][11]. City CO_2 emission was calculated based on the ODIAC dataset[26,27].

Airport code	Latitude (°N)	Longitude (°E)	Elevation (m)	Vertical profile	Flight time (Local Time)	City	Population (thousands)	CO_2 emission ($tC\ yr^{-1}$)
DME	55.409	37.906	179	374	15–20	Moscow	11461	29152105
AMS	52.309	4.764	3	226	14–22	Amsterdam	1057	12408689
LHR	51.477	–0.461	24	264	13–24	London	9699	11864461
YVR	49.194	–123.184	4	431	7–16	Vancouver	2278	2902291
CDG	49.010	2.549	119	661	4–24	Paris	10460	10697964
MXP	45.631	8.728	24	88	17–23	Milano	3056	10779381
CTS	42.775	141.692	25	52	7–22	Sapporo	2591	10584493
BOS	42.363	–71.006	6	83	9–19	Boston	4185	10354860
ORD	41.979	–87.905	205	111	6–13	Chicago	8616	16989467
FCO	41.804	12.251	5	65	17–23	Rome	3592	4209181
JFK	40.640	–73.779	4	118	8–14	New York	18365	23290456
PEK	40.080	116.585	35	78	8–22	Beijing	16190	49682835
SFO	37.619	–122.375	4	378	0–3, 15–16	San Francisco	3283	5369921
ICN	37.469	126.450	7	191	8–22	Incheon	2559	42521466
NRT	35.764	140.392	43	7692	6–23	Tokyo	36834	46603586
HND	35.553	139.781	6	4462	0–24	Tokyo	36834	46603586
NGO	34.858	136.805	5	999	5–23	Nagoya	9165	33924321
ITM	34.785	135.438	12	202	8–21	Osaka	19492	27447862
KIX	34.427	135.244	0	824	7–24	Osaka	19492	27447862
LAX	33.943	–118.408	38	120	8–15	Los Angels	12160	23455740
FUK	33.584	130.452	9	195	7–23	Fukuoka	5556	8057204
SAN	32.734	–117.190	5	54	9–14	San Diego	2964	6715360
SHA	31.198	121.339	3	332	9–14	Shanghai	19980	62338484
DEL	28.566	77.103	237	739	16–24	Delhi	21935	24677421
TPE	25.078	121.233	32	124	9–23	Taipei	2654	17403375
HKG	22.309	113.915	6	812	8–17, 9–24	Hong Kong	7050	36701090
HNL	21.319	–157.922	4	2095	6–14	Honolulu	805	2866534
MEX	19.436	–99.072	2230	55	9–12, 16–18	Mexico City	20132	14557348
MNL	14.509	121.019	23	78	9–16, 21–24	Manila	11891	4942616
BKK	13.681	100.747	2	1670	0–24	Bangkok	8213	18910974
GUM	13.483	144.796	91	66	10–15, 21–27	Guam	143	1049821
SIN	1.350	103.994	7	930	8–10, 16–19, 22–25	Singapore	5079	27456795
CGK	–6.126	106.656	10	493	16–18, 21–24	Jakarta	9630	16197473
DPS	–8.748	115.168	4	66	21–26	Denpasar	797	2463670
BNE	–27.384	153.118	4	96	6–11	Brisbane	2034	6893168
SYD	–33.946	151.177	6	1568	5–11	Sydney	4364	6126149

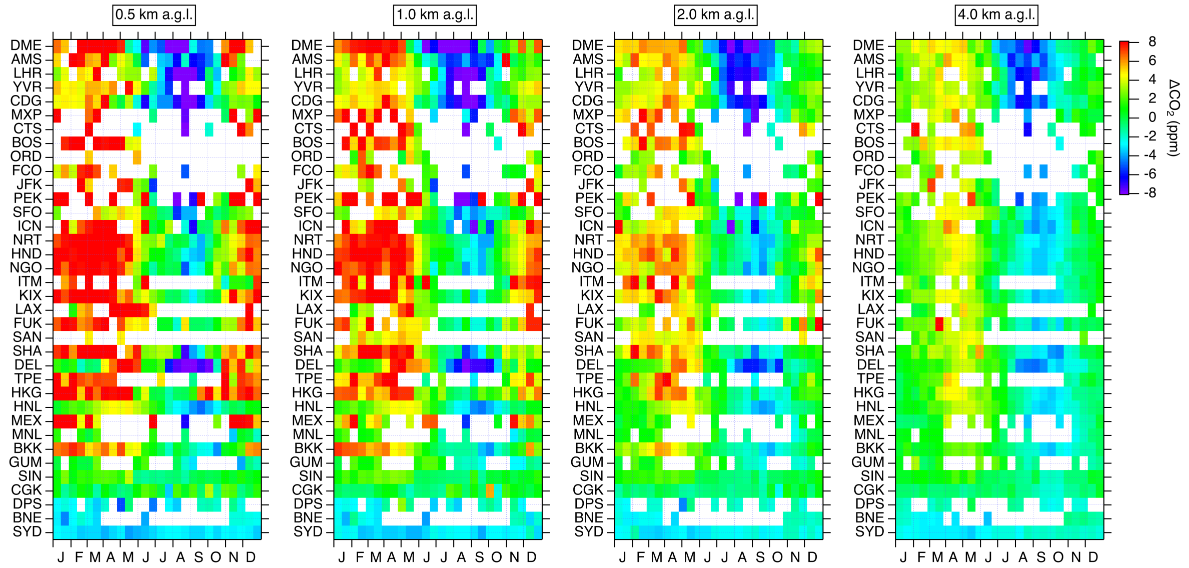

Supplementary Figure S1. Seasonal climatology of ΔCO_2 (median) at different altitude bins (above ground level, a.g.l.) over different airports. See Supplementary Table S1 for airport codes.

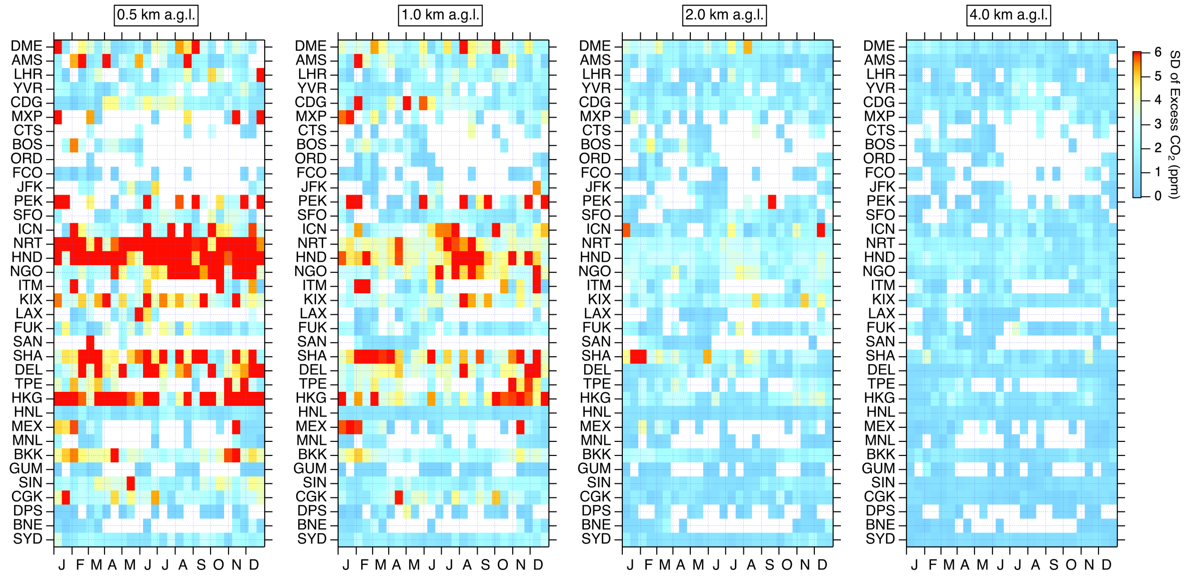

Supplementary Figure S2. Seasonal climatology of standard deviation (SD) of excess CO_2 at different altitude bins over different airports. See Supplementary Table 1 for airport codes.

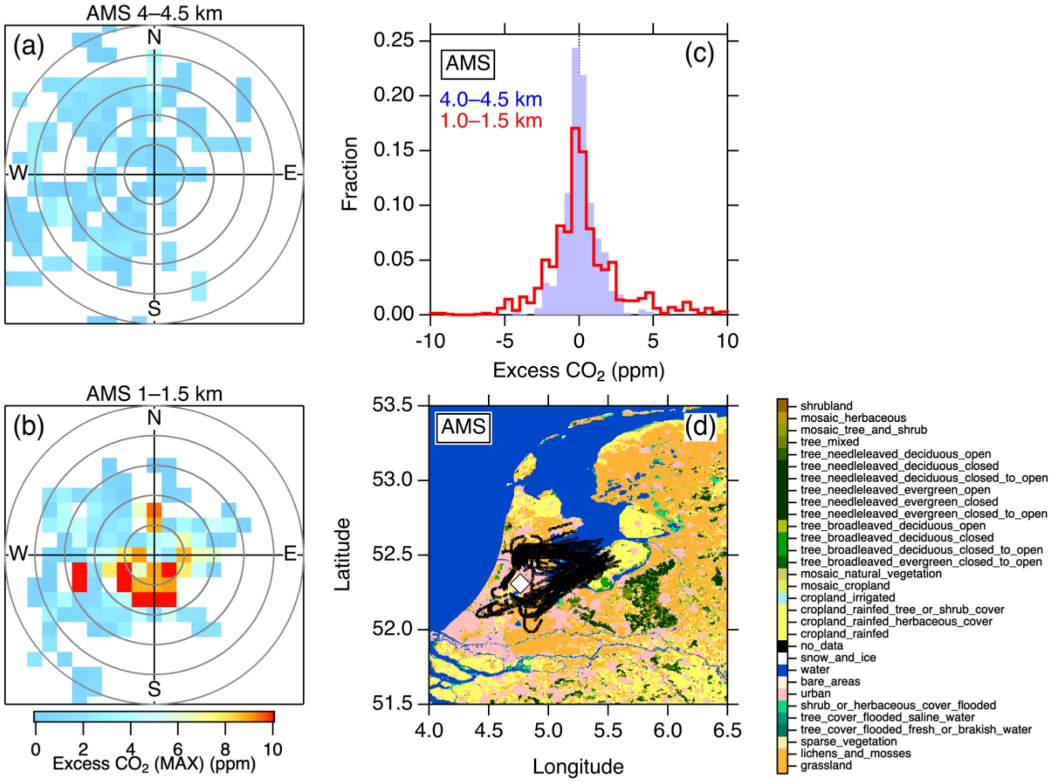

Supplementary Figure S3. Same as Fig. 1, but for Amsterdam Airport Schiphol (AMS).

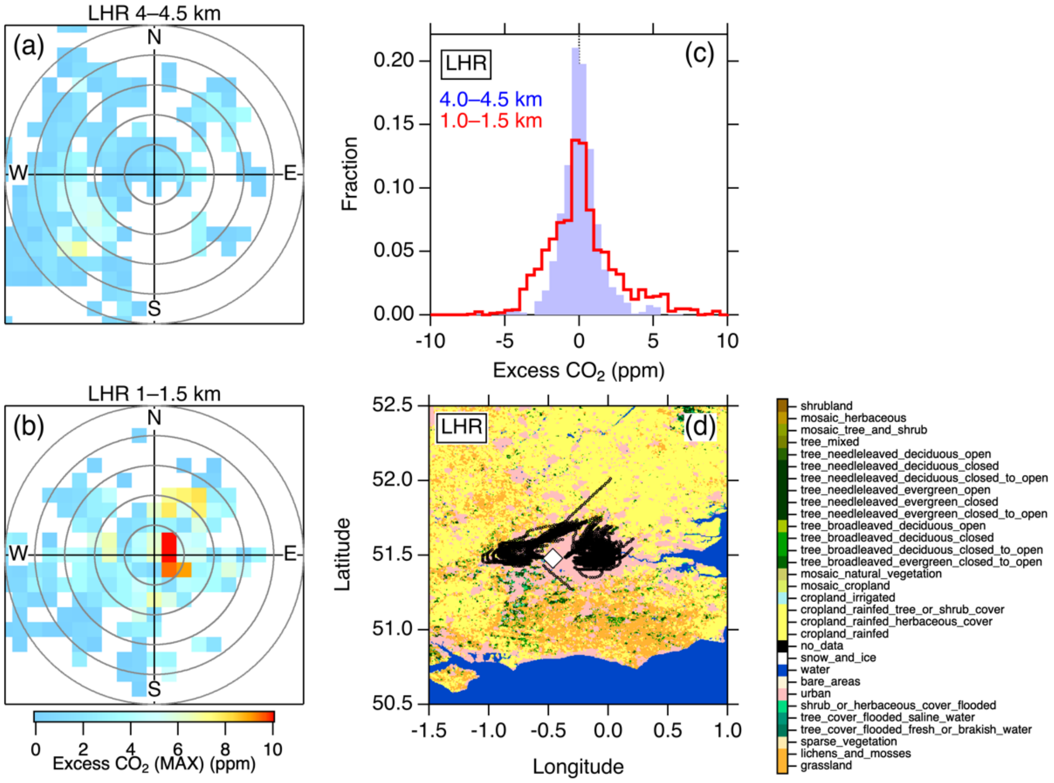

Supplementary Figure S4. Same as Fig. 1, but for London Heathrow Airport (LHR).

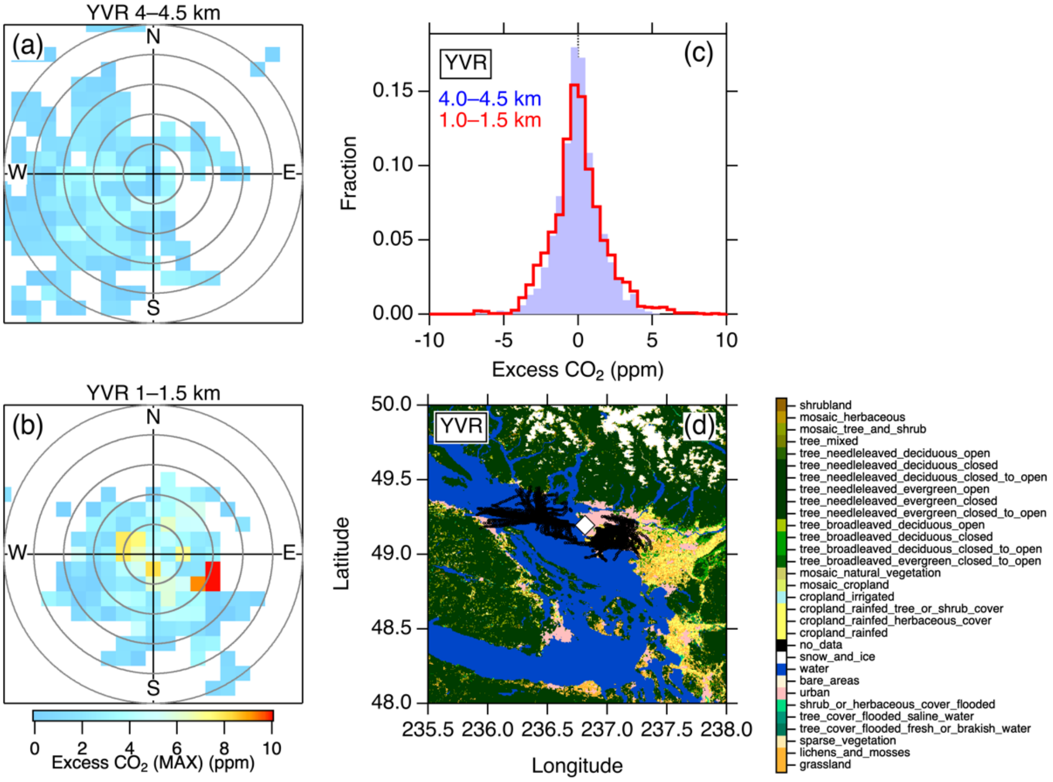

Supplementary Figure S5. Same as Fig. 1, but for Vancouver International Airport (YVR).

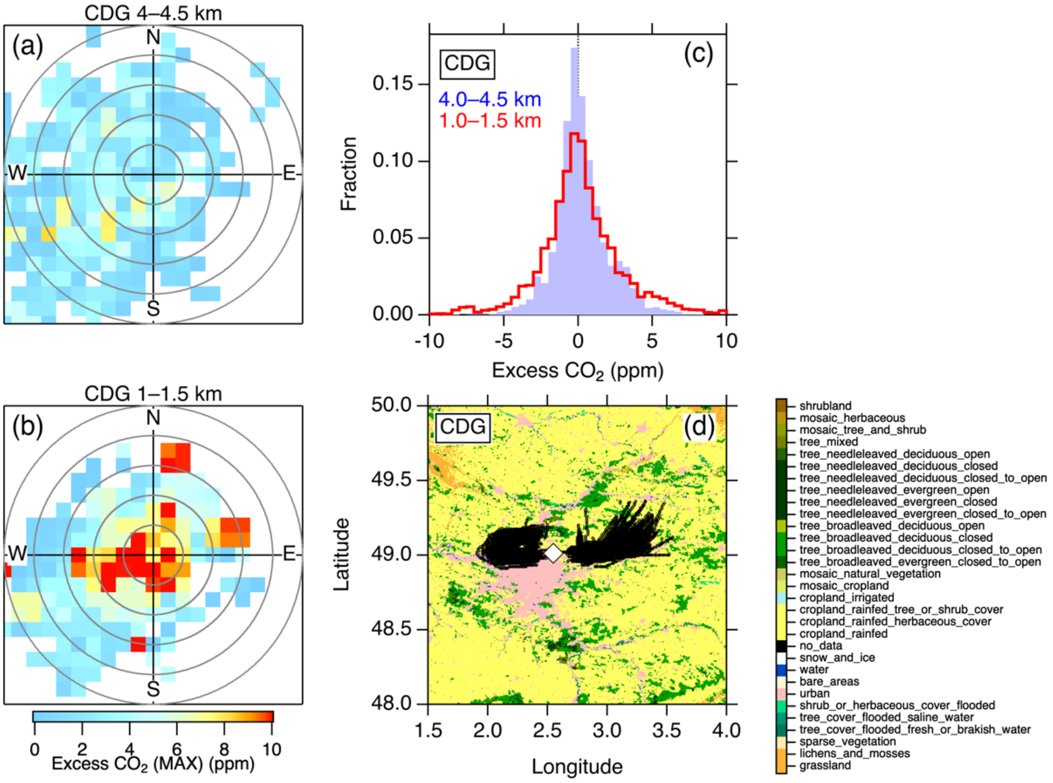

Supplementary Figure S6. Same as Fig. 1, but for Paris Charles de Gaulle Airport (CDG).

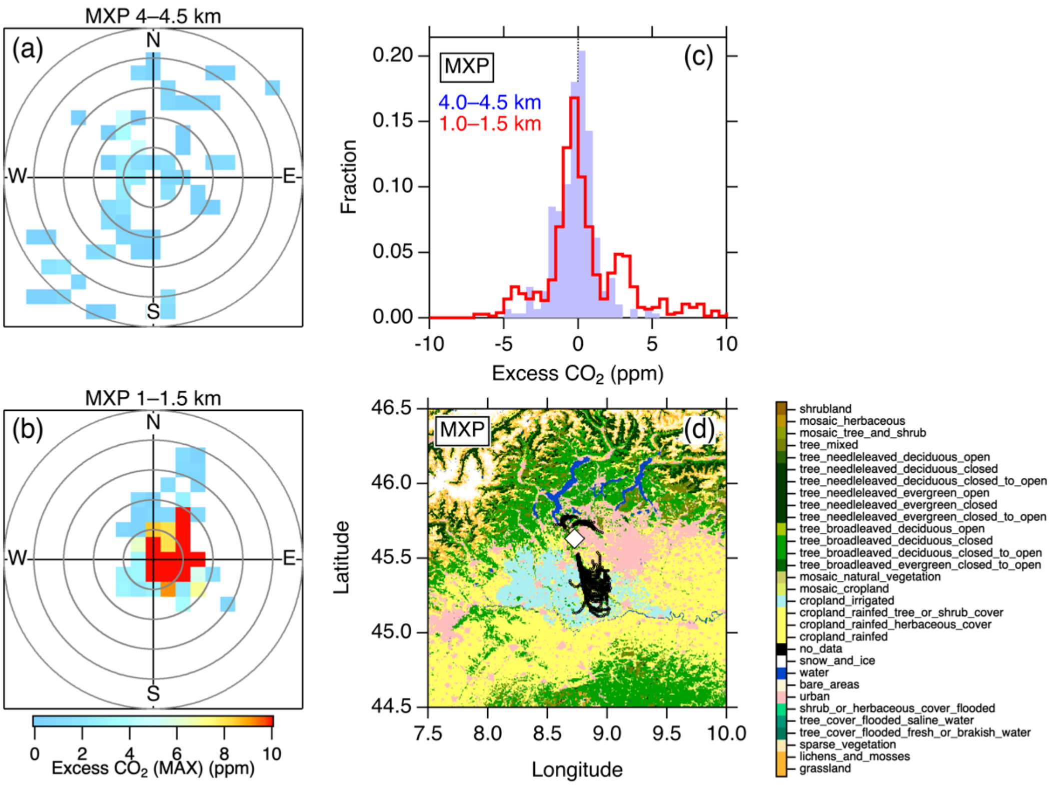

Supplementary Figure S7. Same as Fig. 1, but for Milano Malpensa Airport (MXP).

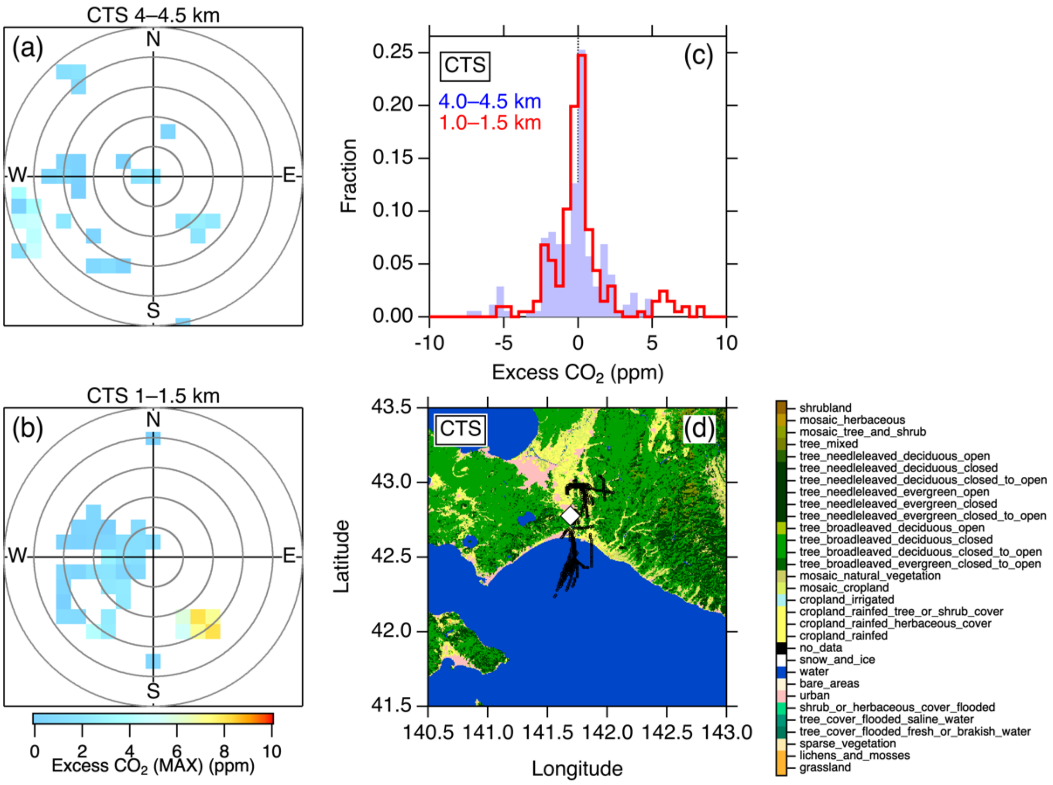

Supplementary Figure S8. Same as Fig. 1, but for New Chitose Airport (CTS).

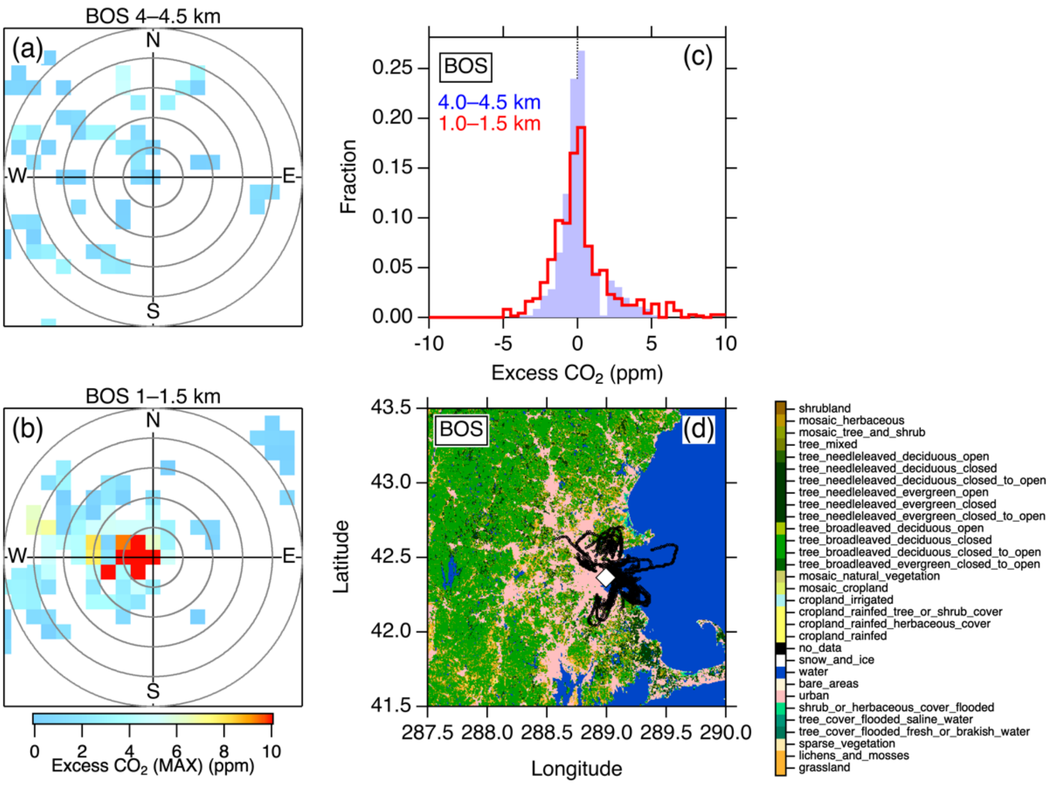

Supplementary Figure S9. Same as Fig. 1, but for Boston Logan International Airport (BOS).

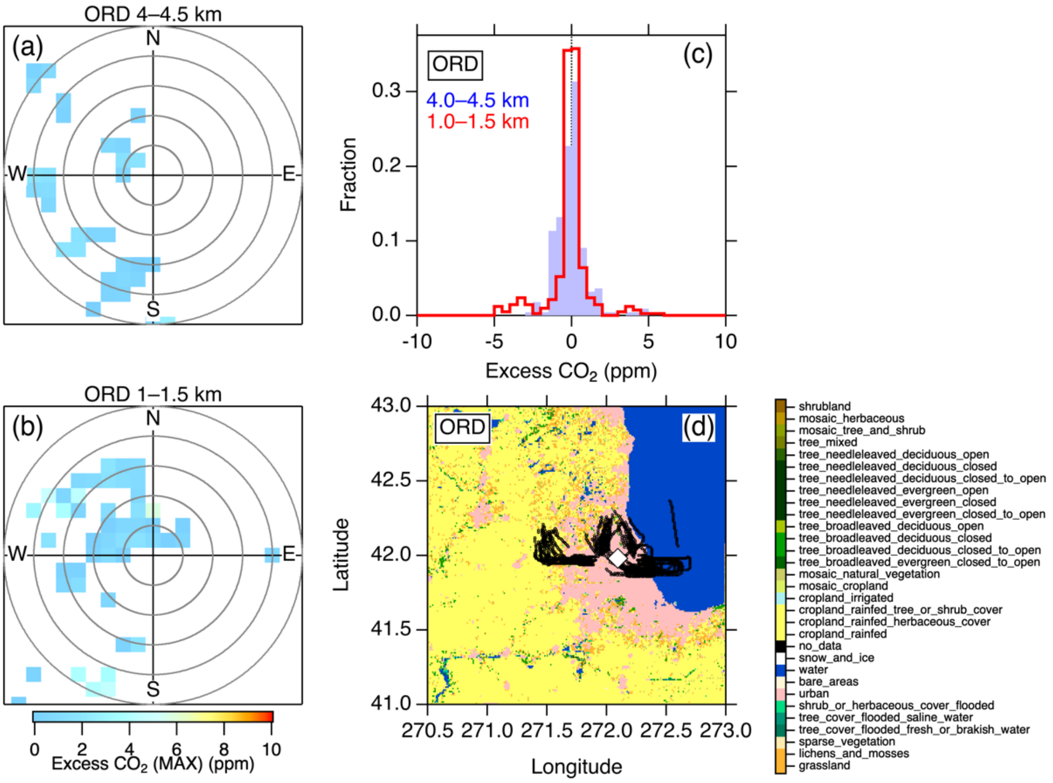

Supplementary Figure S10. Same as Fig. 1, but for Chicago O'Hare International Airport (ORD).

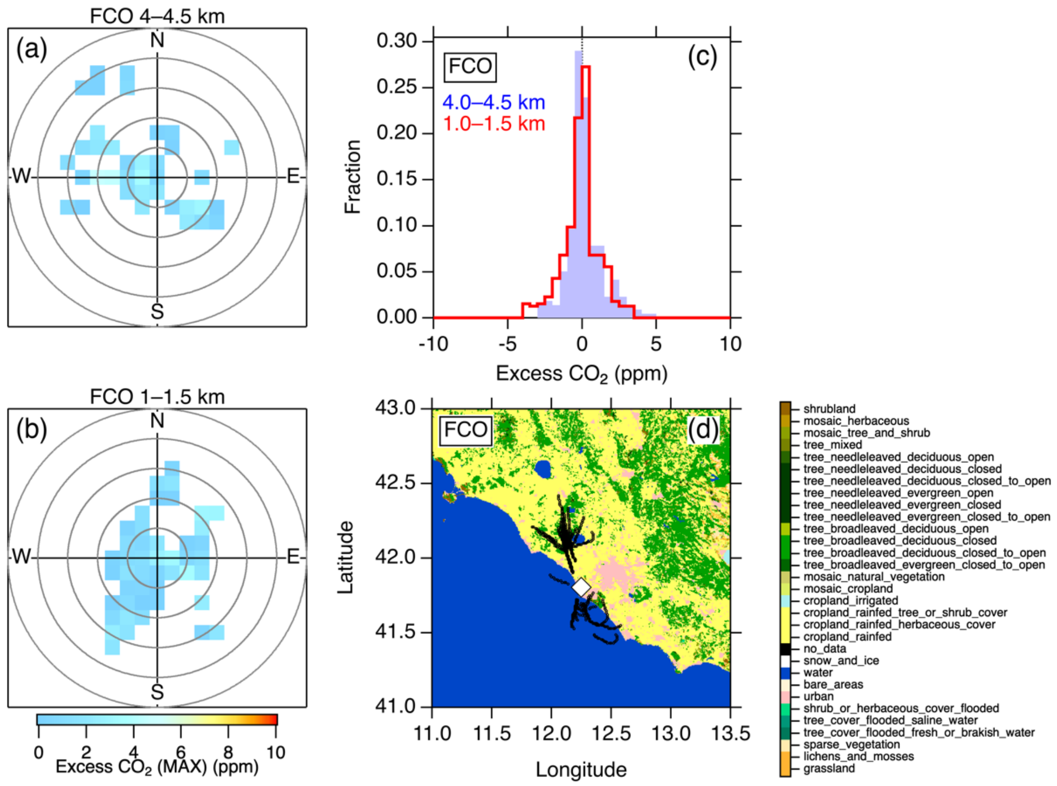

Supplementary Figure S11. Same as Fig. 1, but for Rome–Fiumicino International Airport (FCO).

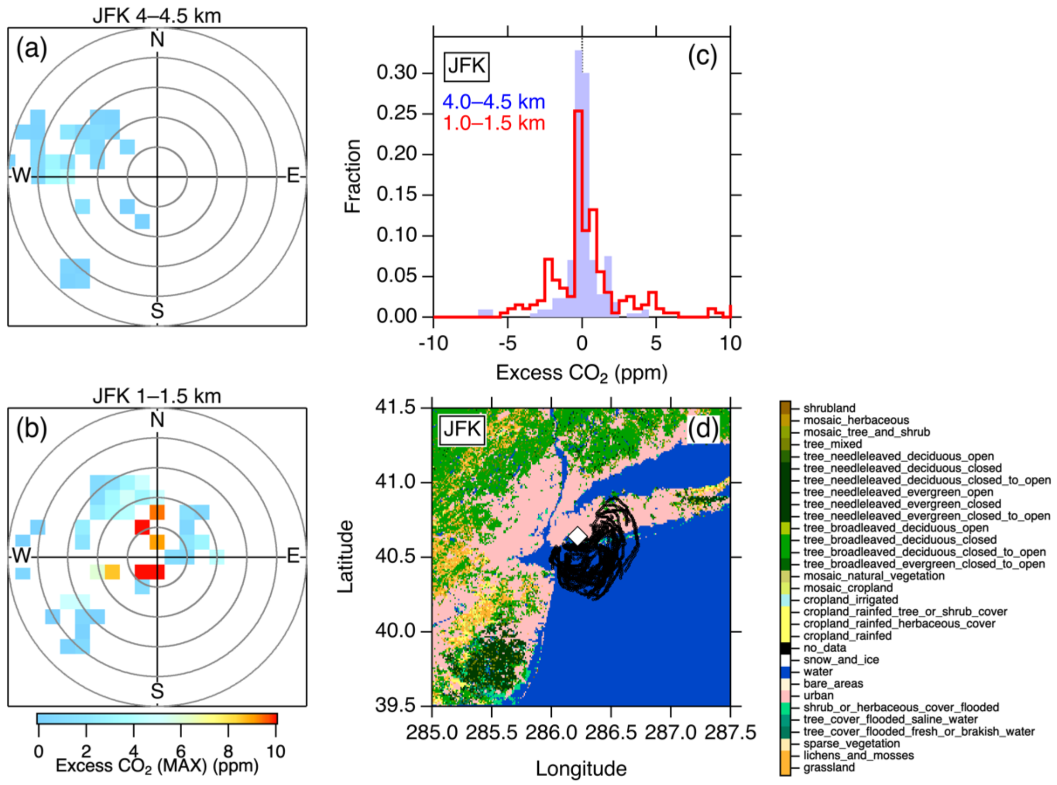

Supplementary Figure S12. Same as Fig. 1, but for John F. Kennedy International Airport (JFK).

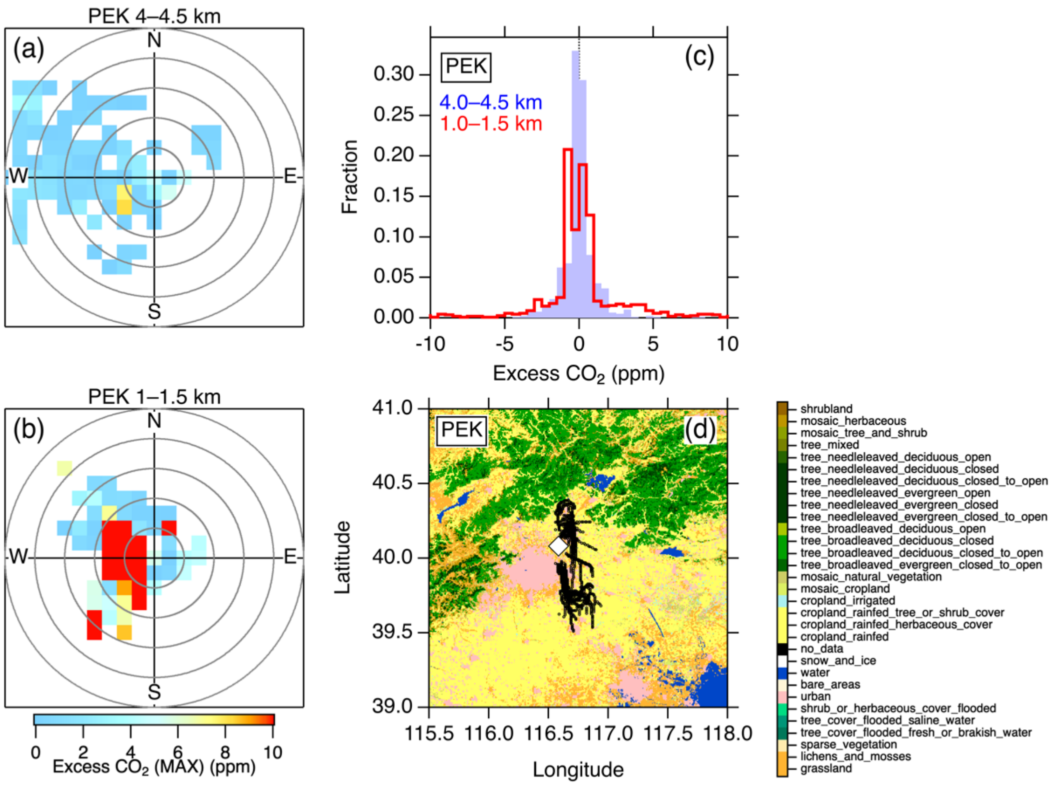

Supplementary Figure S13. Same as Fig. 1, but for Beijing Capital International Airport (PEK).

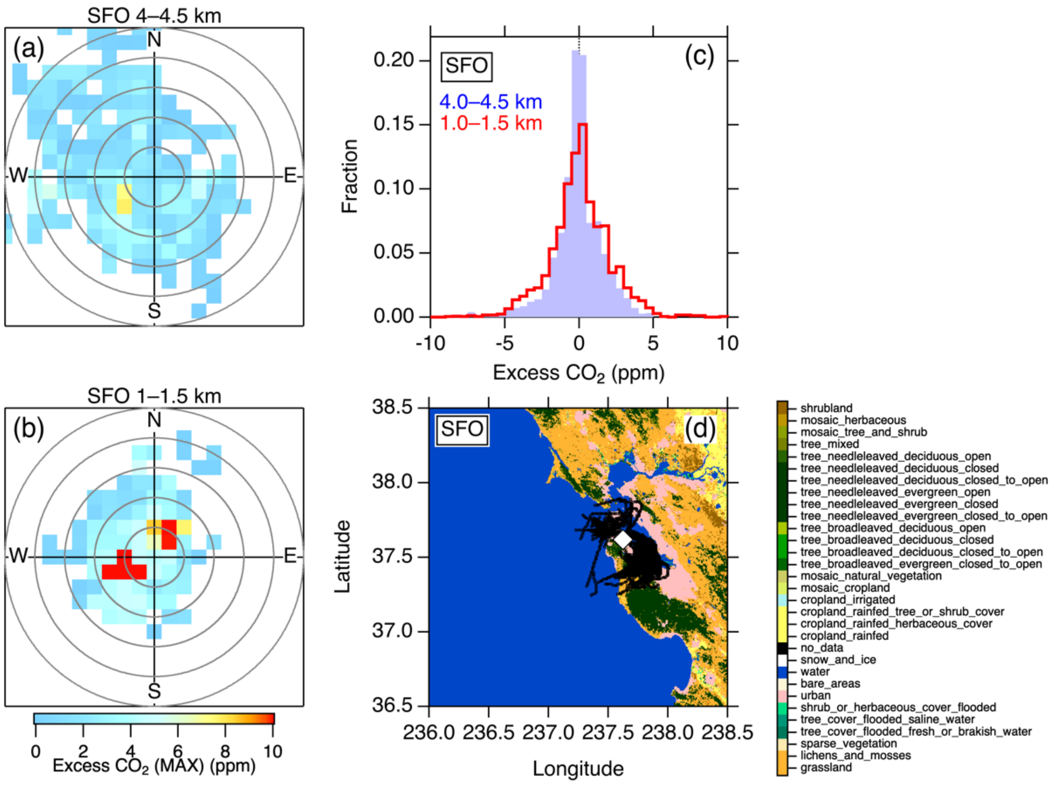

Supplementary Figure S14. Same as Fig. 1, but for San Francisco International Airport (SFO).

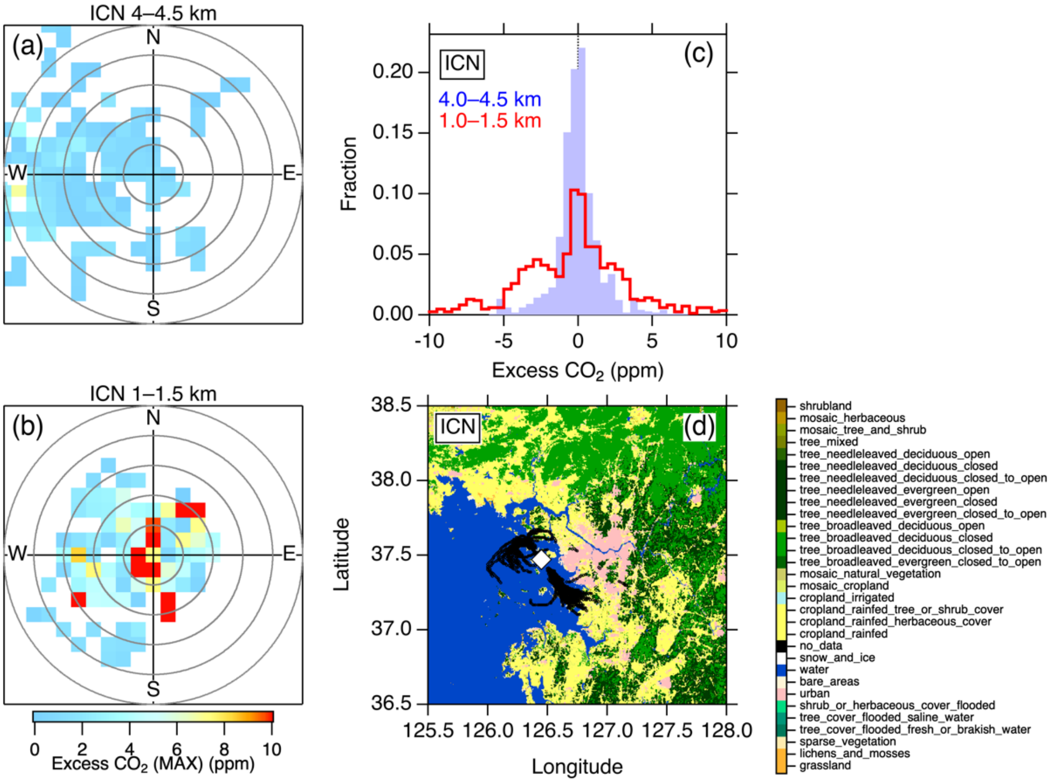

Supplementary Figure S15. Same as Fig. 1, but for Incheon International Airport (ICN).

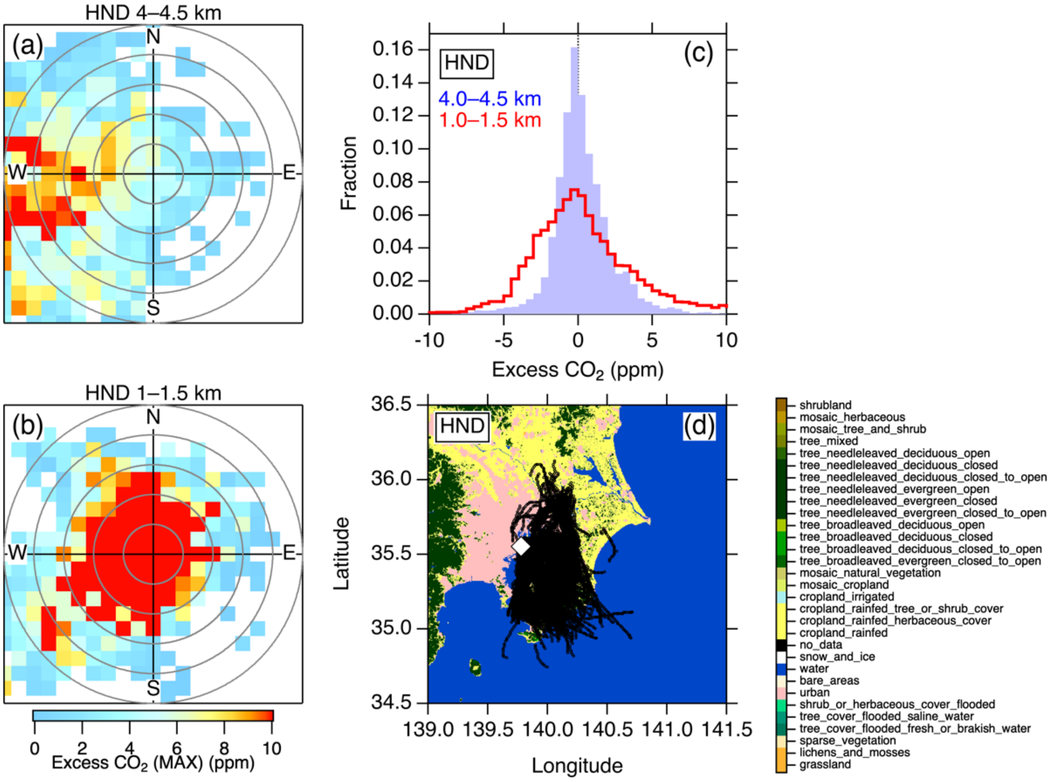

Supplementary Figure S16. Same as Fig. 1, but for Tokyo International Airport (HND).

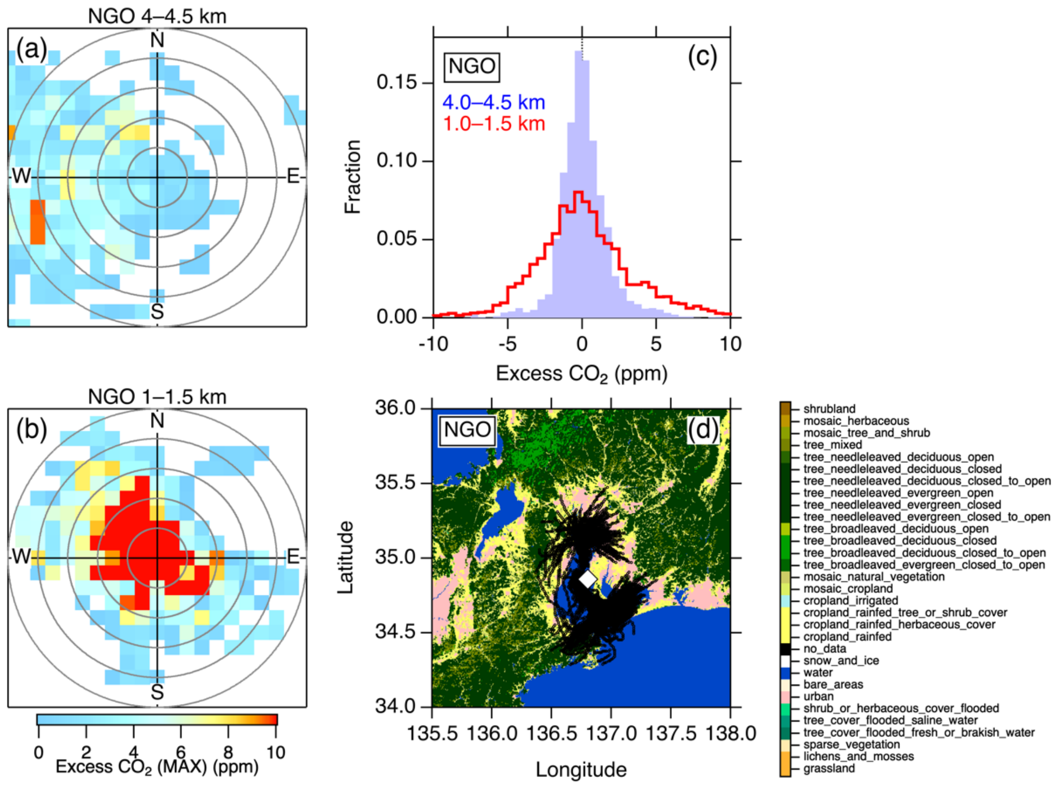

Supplementary Figure S17. Same as Fig. 1, but for Chubu Centrair International Airport (NGO).

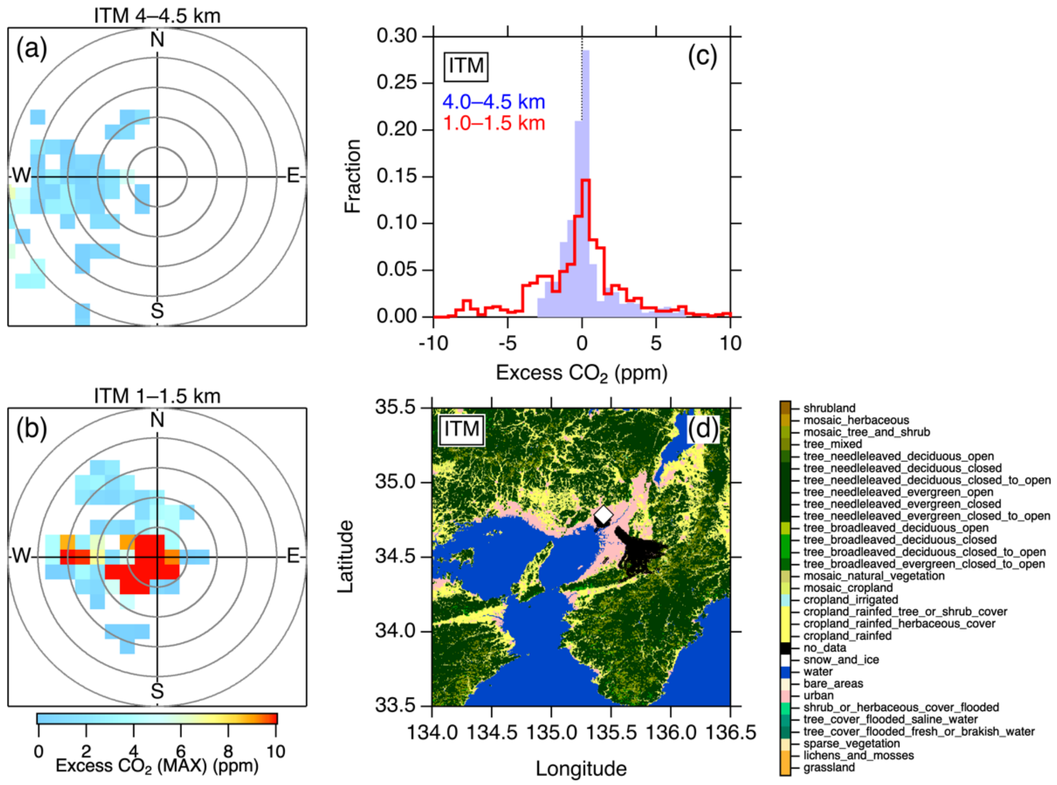

Supplementary Figure S18. Same as Fig. 1, but for Osaka International Airport (ITM).

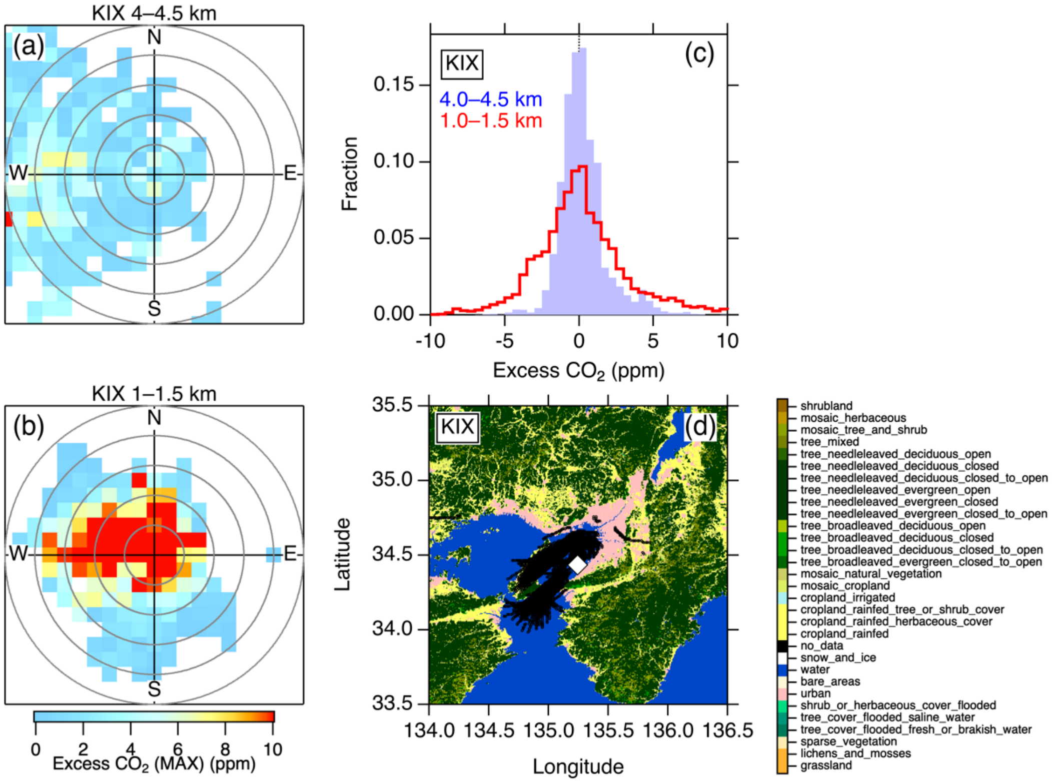

Supplementary Figure S19. Same as Fig. 1, but for Kansai International Airport (KIX).

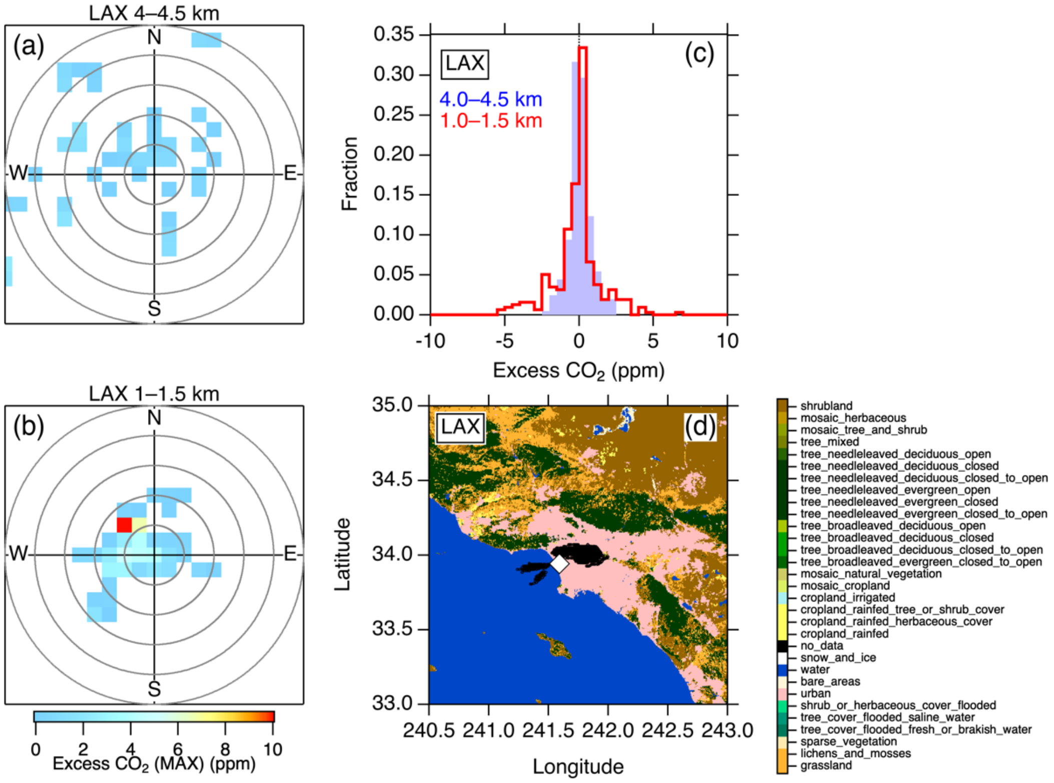

Supplementary Figure S20. Same as Fig. 1, but for Los Angeles International Airport (LAX).

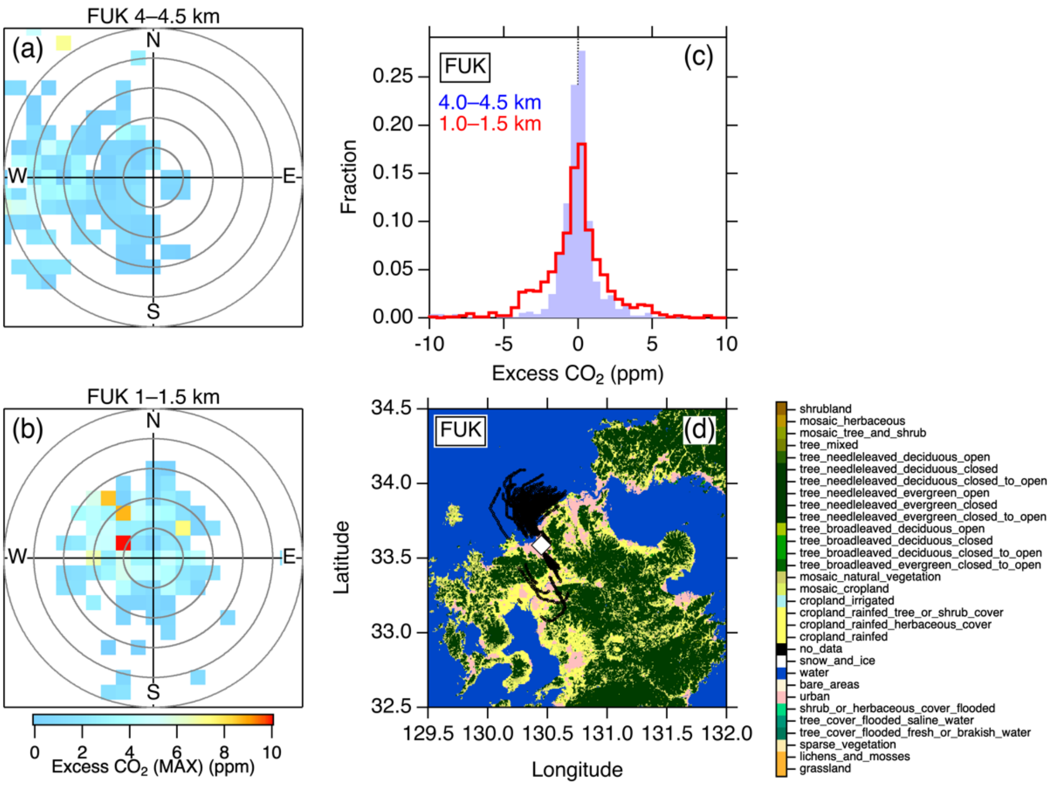

Supplementary Figure S21. Same as Fig. 1, but for Fukuoka Airport (FUK).

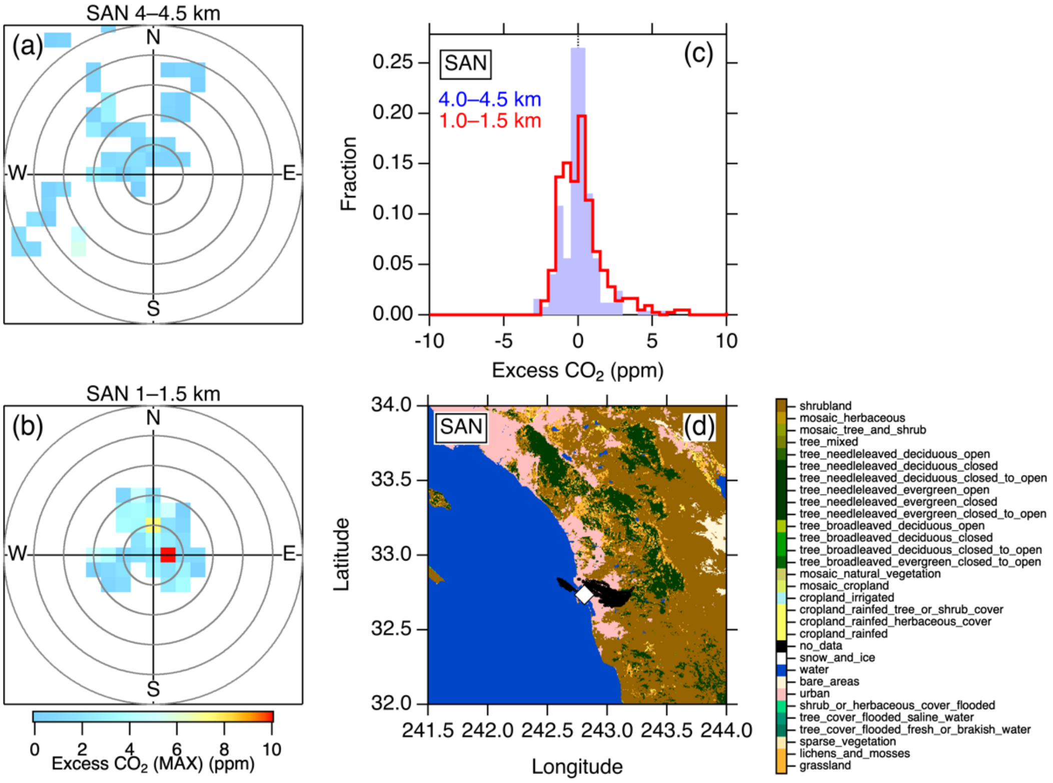

Supplementary Figure S22. Same as Fig. 1, but for San Diego International Airport (SAN).

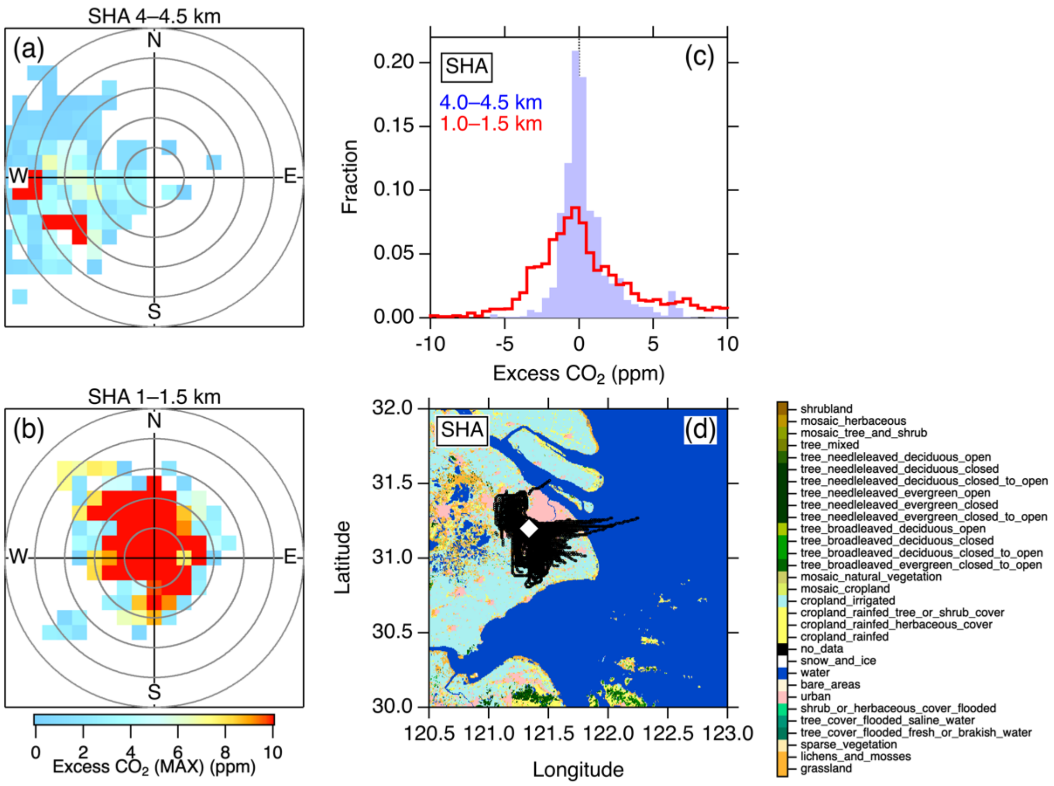

Supplementary Figure S23. Same as Fig. 1, but for Shanghai Hongqiao International Airport (SHA).

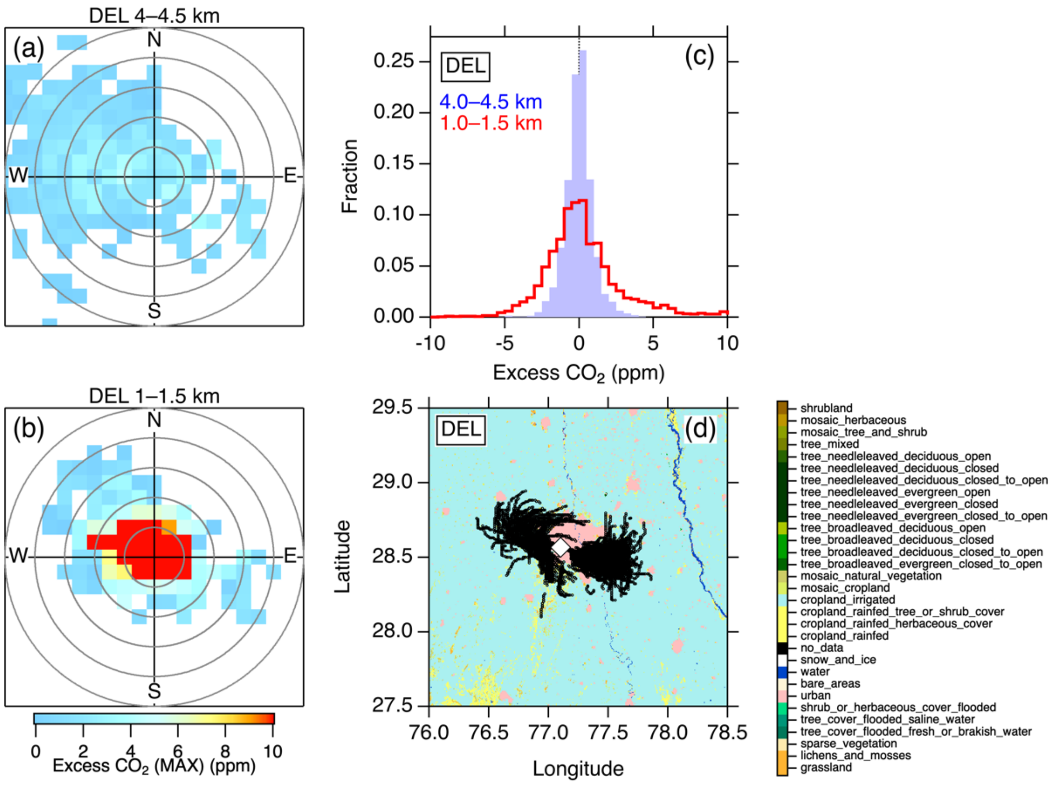

Supplementary Figure S24. Same as Fig. 1, but for Indira Gandhi International Airport (DEL).

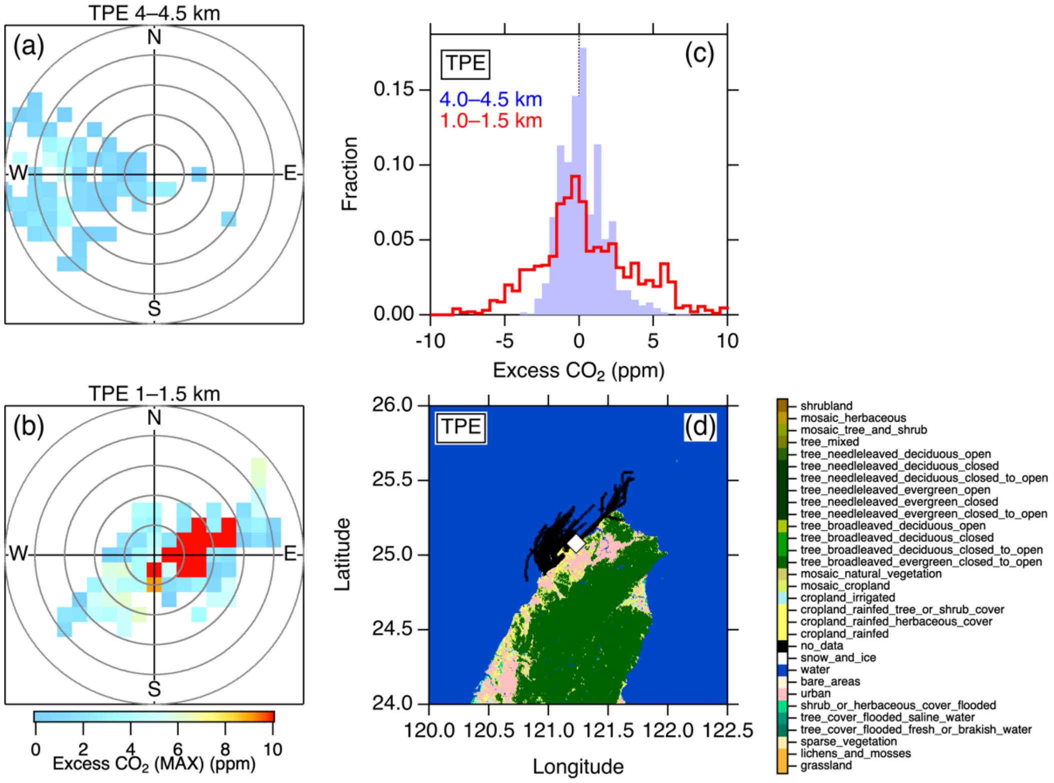

Supplementary Figure S25. Same as Fig. 1, but for Taiwan Taoyuan International Airport (TPE).

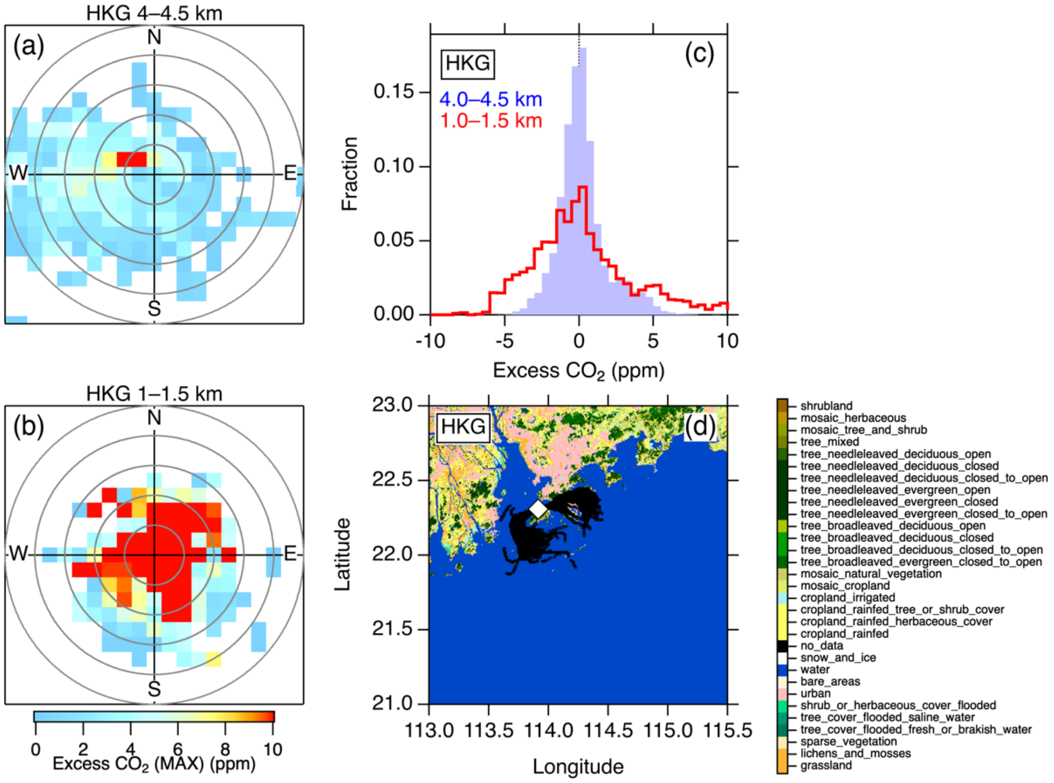

Supplementary Figure S26. Same as Fig. 1, but for Hong Kong International Airport (HKG).

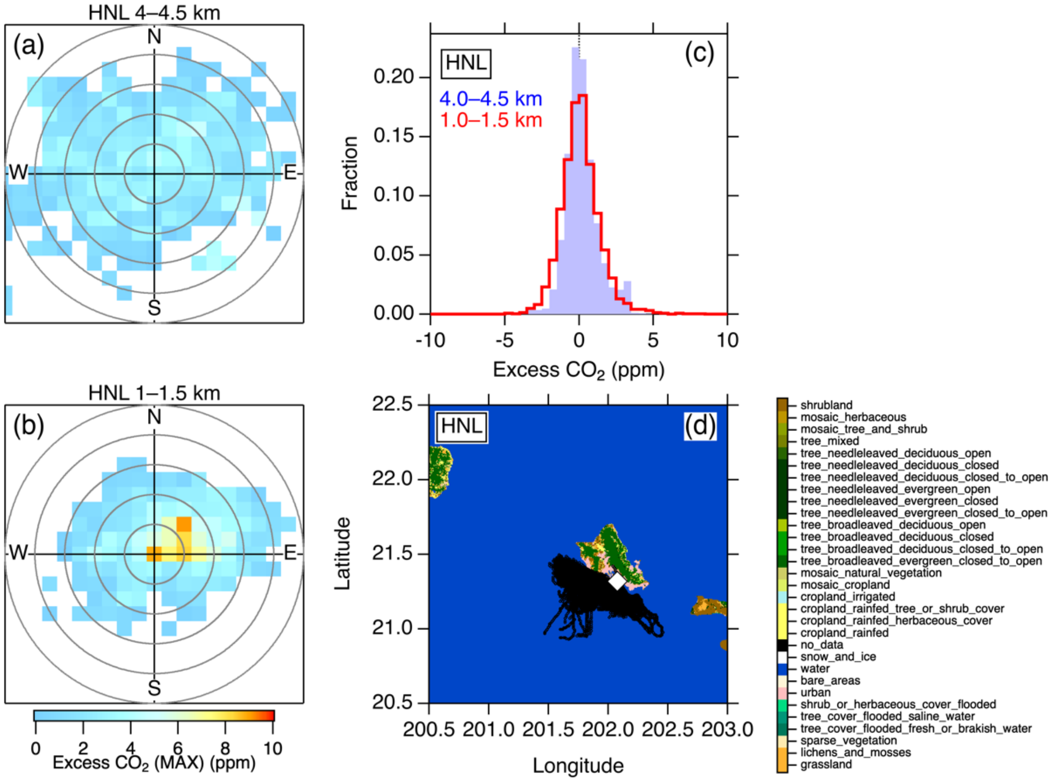

Supplementary Figure S27. Same as Fig. 1, but for Daniel K. Inouye International Airport (HNL).

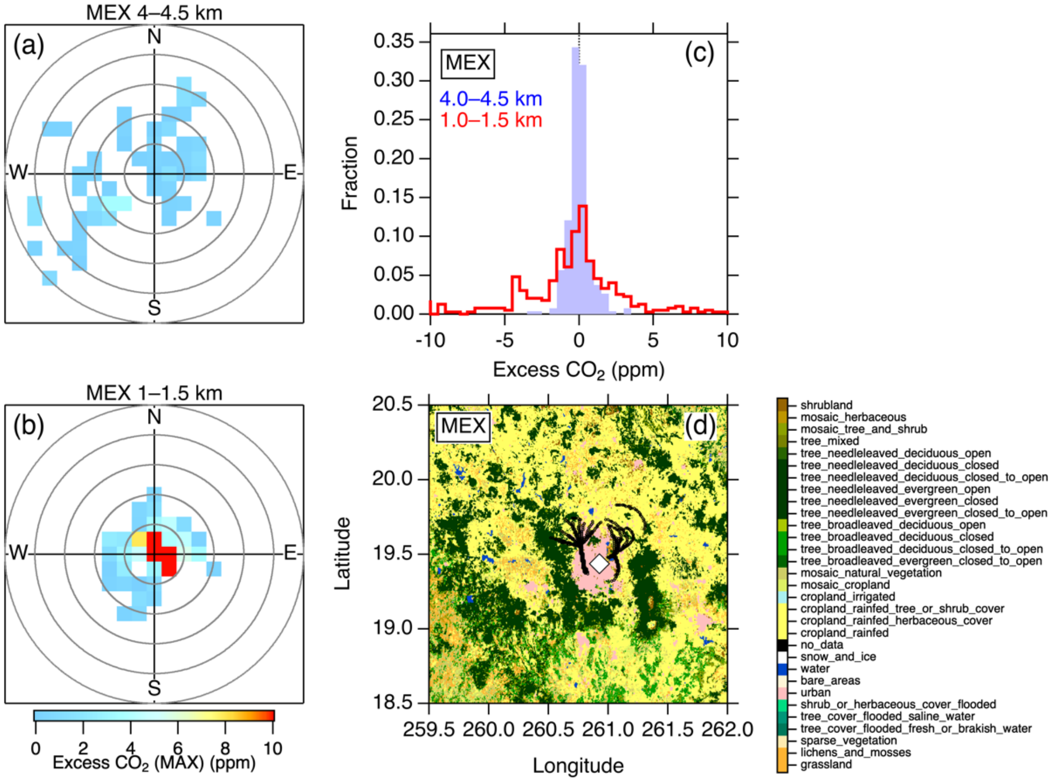

Supplementary Figure S28. Same as Fig. 1, but for Mexico City International Airport (MEX).

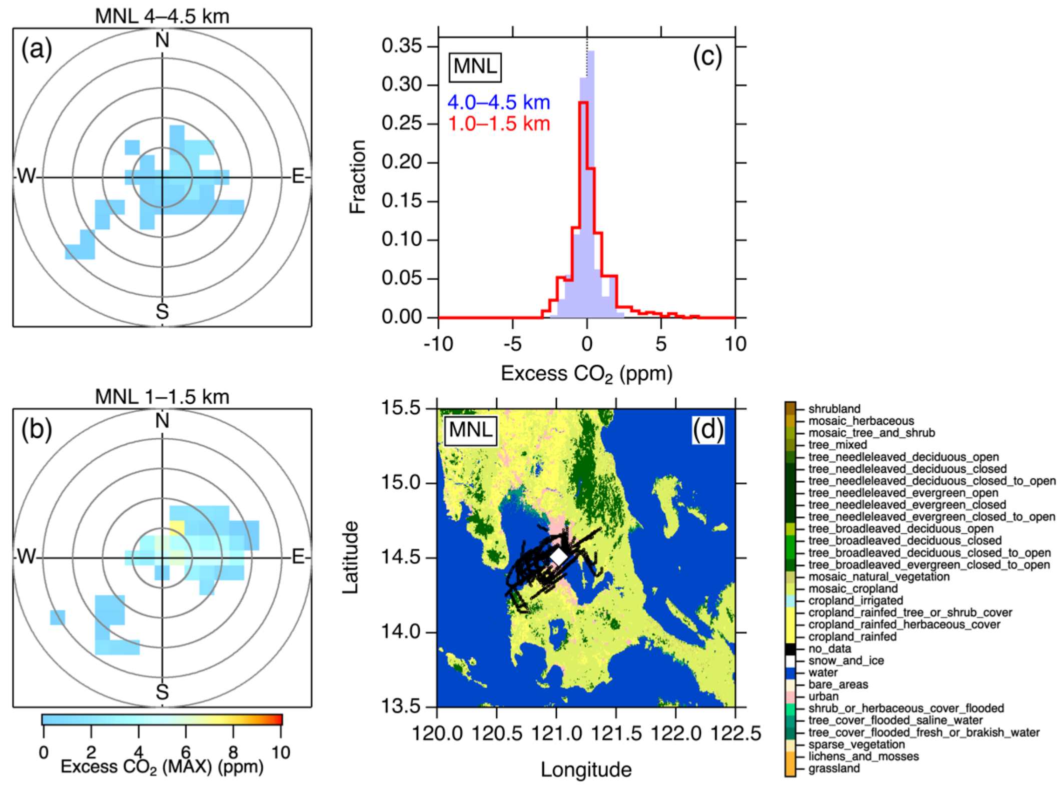

Supplementary Figure S29. Same as Fig. 1, but for Ninoy Aquino International Airport (MNL).

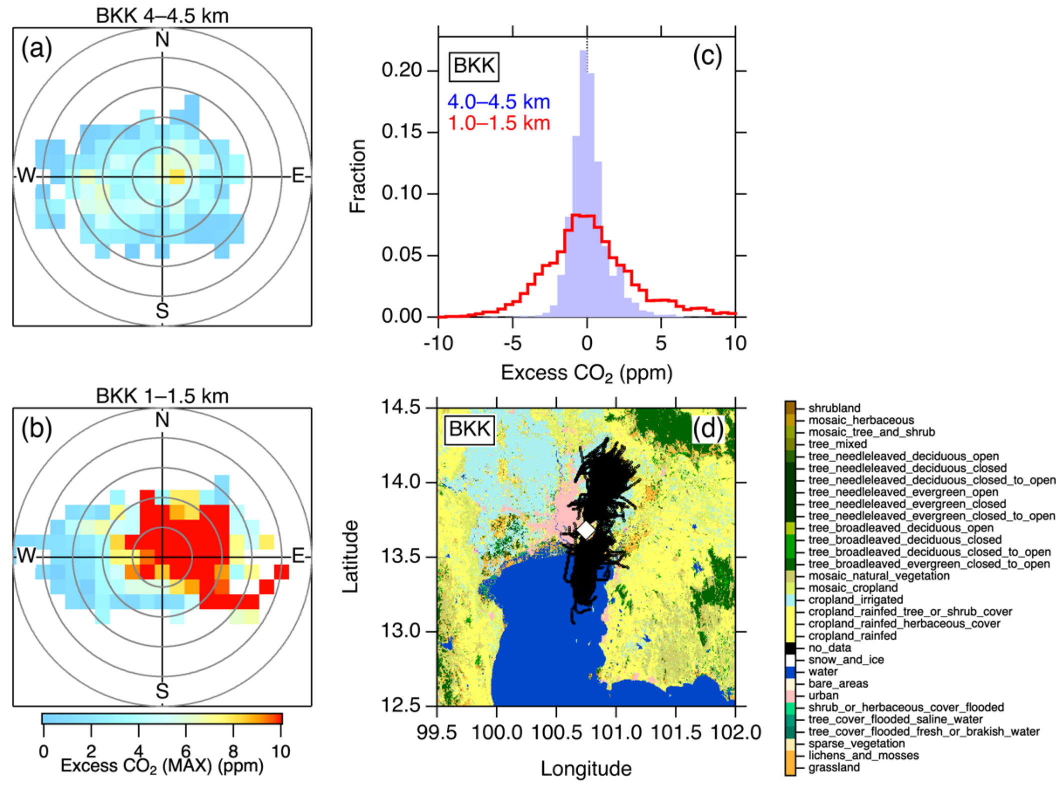

Supplementary Figure S30. Same as Fig. 1, but for Suvarnabhumi Airport (BKK).

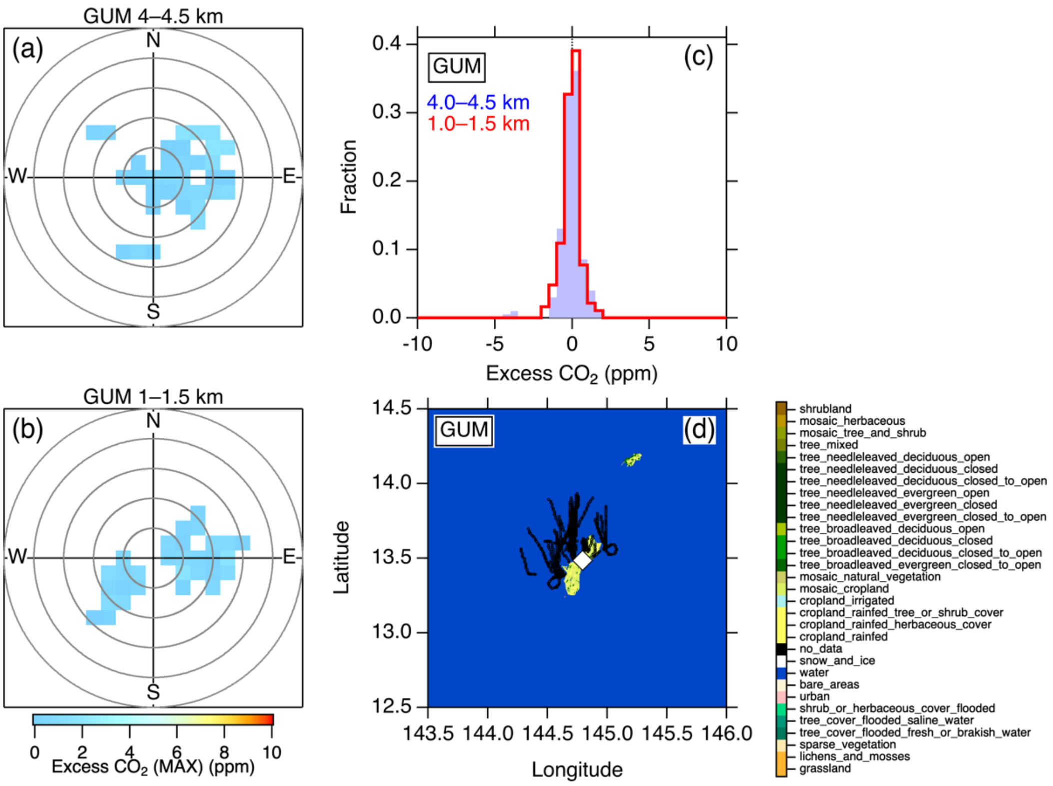

Supplementary Figure S31. Same as Fig. 1, but for Antonio B. Won Pat International Airport (GUM).

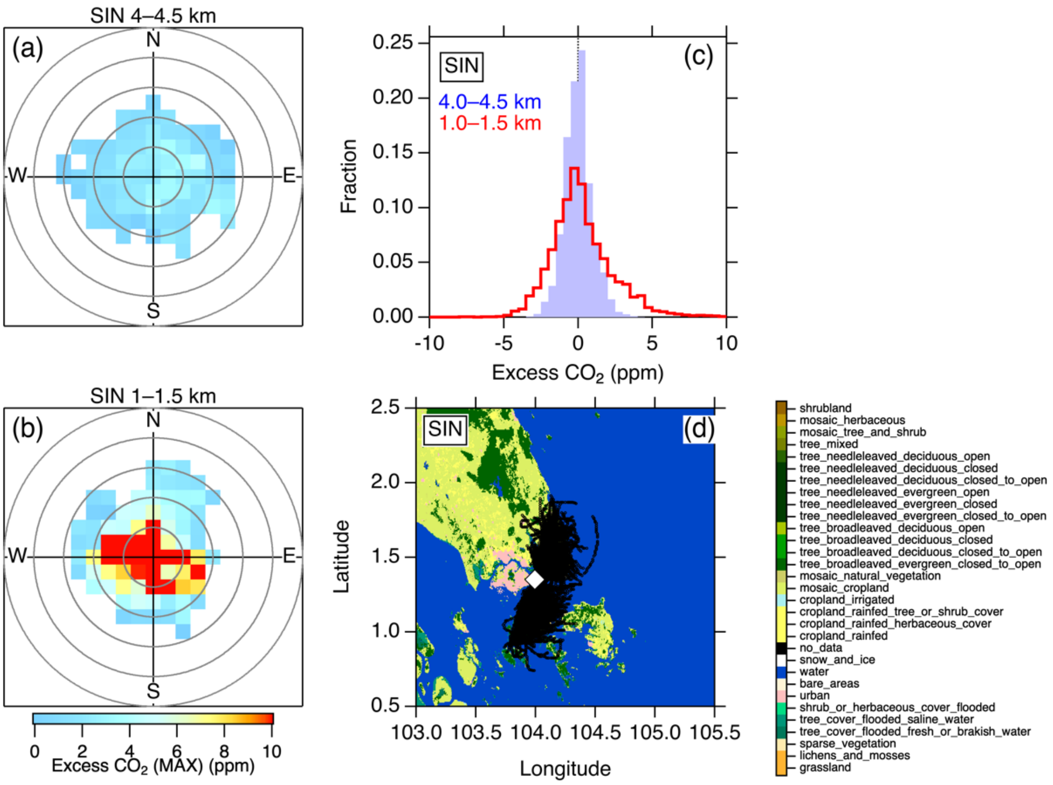

Supplementary Figure S32. Same as Fig. 1, but for Singapore Changi Airport (SIN).

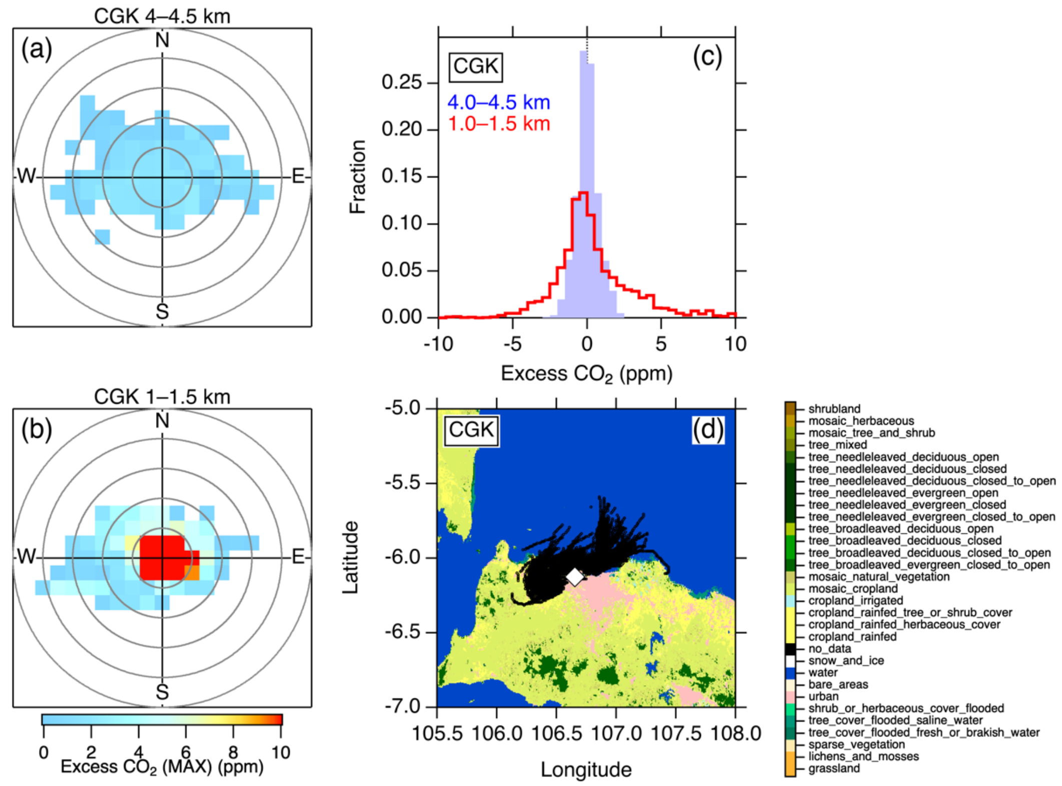

Supplementary Figure S33. Same as Fig. 1, but for Soekarno–Hatta International Airport (CGK).

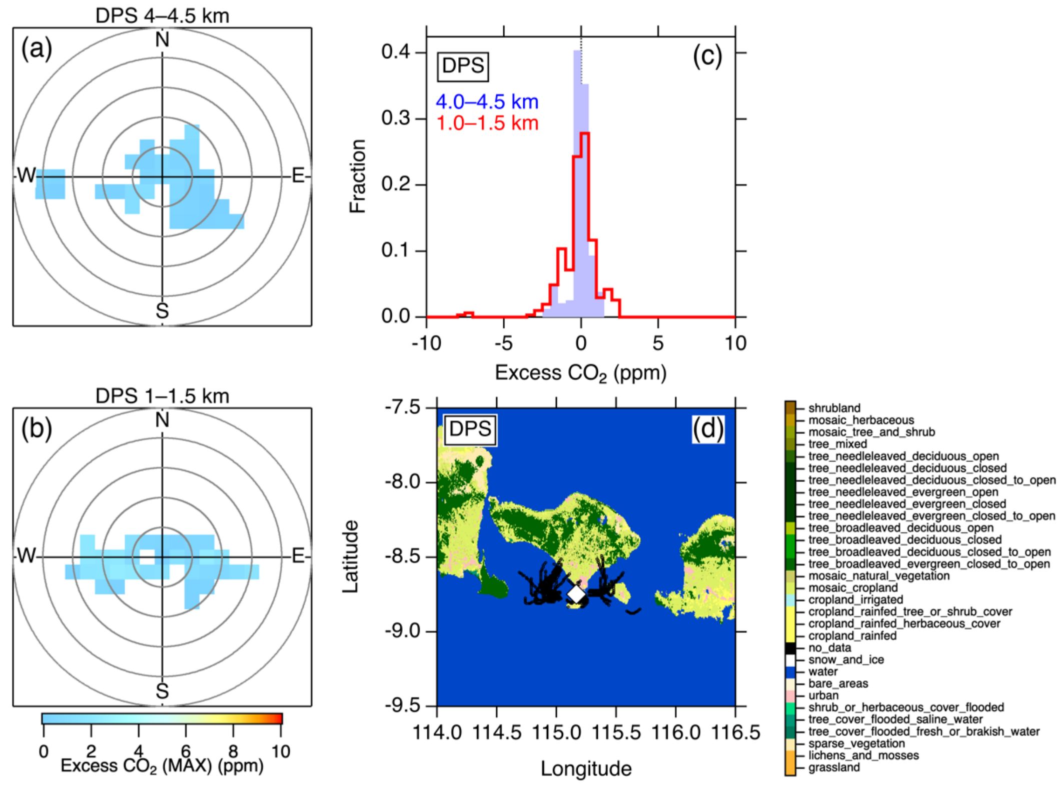

Supplementary Figure S34. Same as Fig. 1, but for I Gusti Ngurah Rai International Airport (DPS).

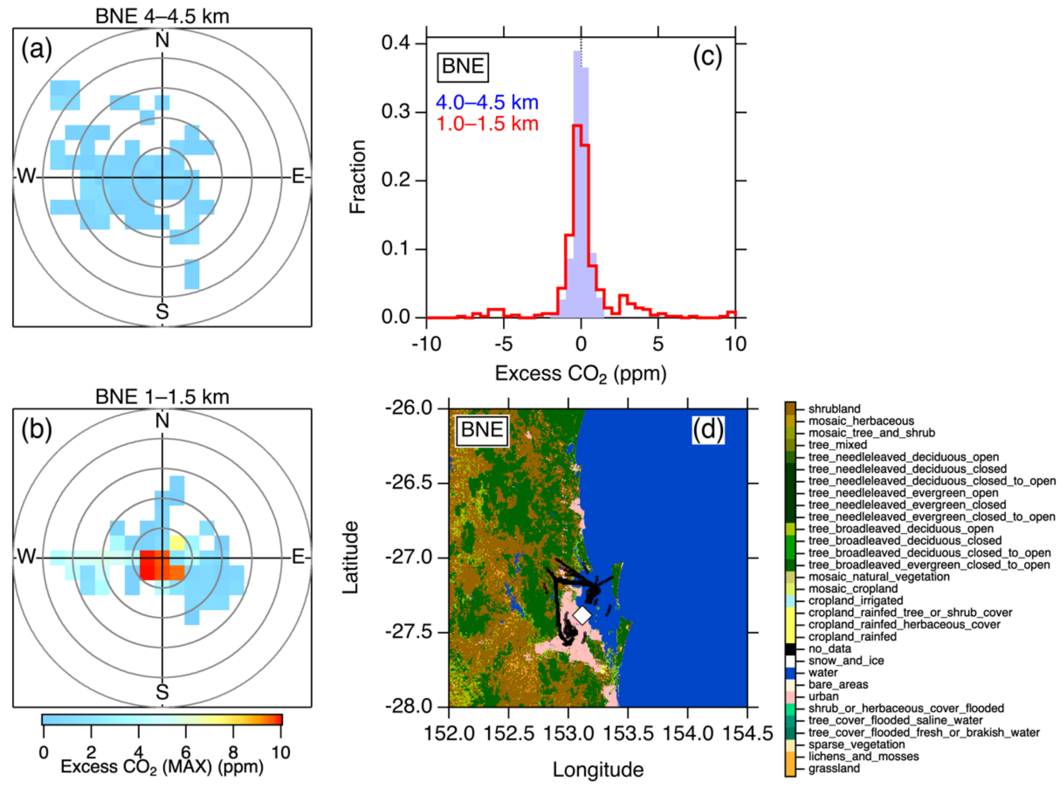

Supplementary Figure S35. Same as Fig. 1, but for Brisbane Airport (BNE).

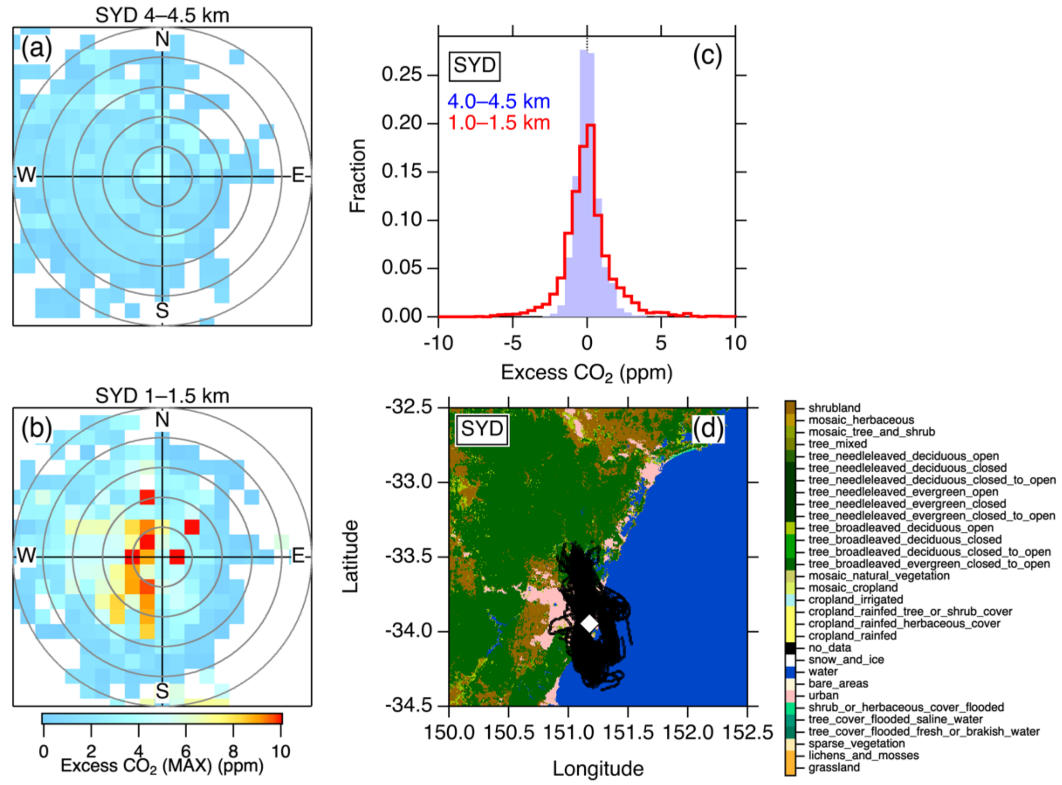

Supplementary Figure S36. Same as Fig. 1, but for Sydney (Kingsford Smith) Airport (SYD).

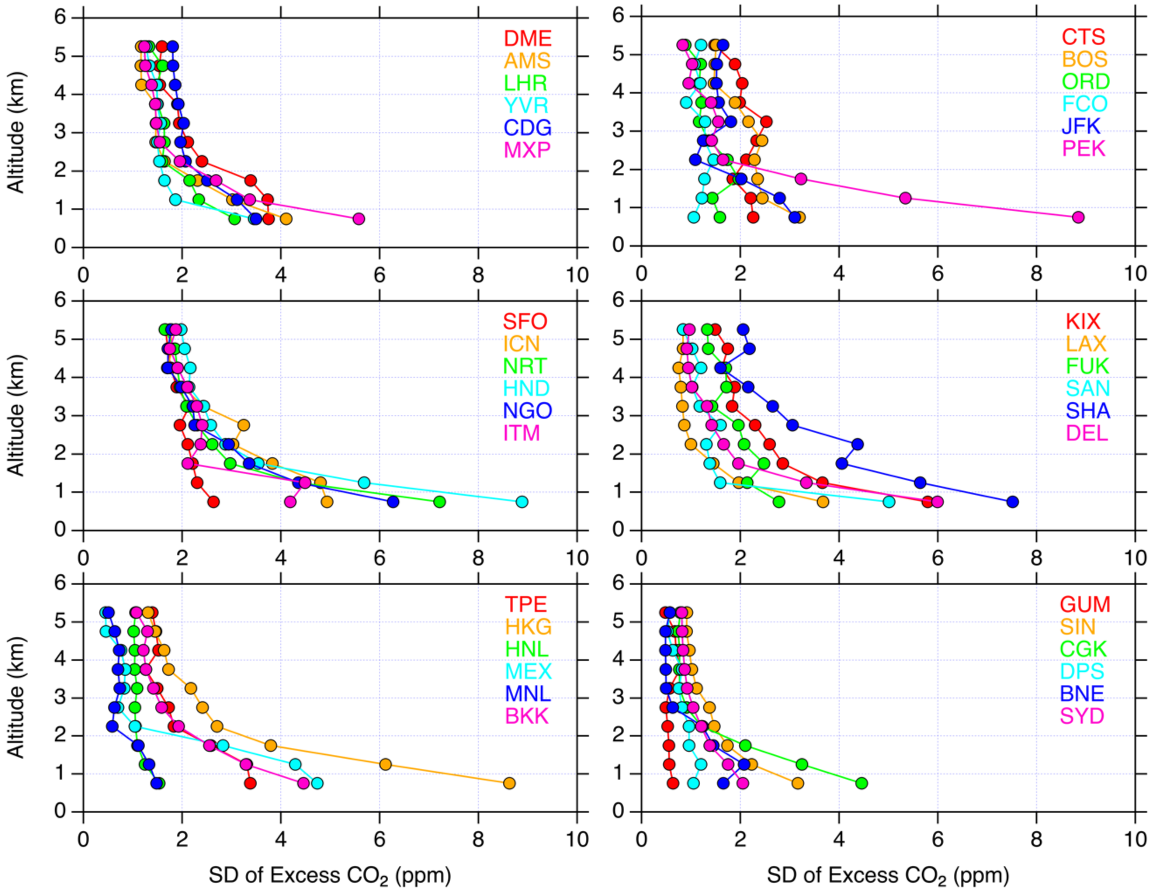

Supplementary Figure S37. Vertical profiles of standard deviations of excess CO_2 at the 36 airports.
